# Fluorination Influences the Bioisostery of *Myo*-Inositol Pyrophosphate Analogs

**DOI:** 10.1002/chem.202302426

**Published:** 2023-10-19

**Authors:** Sarah Hostachy, Huanchen Wang, Guangning Zong, Katy Franke, Andrew M. Riley, Peter Schmieder, Barry V. L. Potter, Stephen B. Shears, Dorothea Fiedler

**Affiliations:** a Leibniz-Forschungsinstitut für Molekulare Pharmakologie (FMP) Robert-Rössle-Straße 10, 13125 Berlin, Germany; b Inositol Signaling Group National Institutes of Health, Research Triangle Park, North Carolina, 27709, USA; c Medicinal Chemistry & Drug Discovery, Department of Pharmacology University of Oxford, Oxford OX1 3QT, United Kingdom; d Institut für Chemie Humboldt-Universität zu Berlin Brook-Taylor-Str. 2, 12489 Berlin, Germany

**Keywords:** fluorine, inositol pyrophosphates, phosphoramidite, phosphorylation, protein structures

## Abstract

Inositol pyrophosphates (PP-IPs) are densely phosphorylated messenger molecules involved in numerous biological processes. PP-IPs contain one or two pyrophosphate group(s) attached to a phosphorylated myo-inositol ring. 5PP-IP5 is the most abundant PP-IP in human cells. To investigate the function and regulation by PP-IPs in biological contexts, metabolically stable analogs have been developed. Here, we report the synthesis of a new fluorinated phosphoramidite reagent and its application for the synthesis of a difluoromethylene bisphosphonate analog of 5PP-IP5. Subsequently, the properties of all currently reported analogs were benchmarked using a number of biophysical and biochemical methods, including co-crystallization, ITC, kinase activity assays and chromatography. Together, the results showcase how small structural alterations of the analogs can have notable effects on their properties in a biochemical setting and will guide in the choice of the most suitable analog(s) for future investigations.

## Introduction

**Figure 1:**
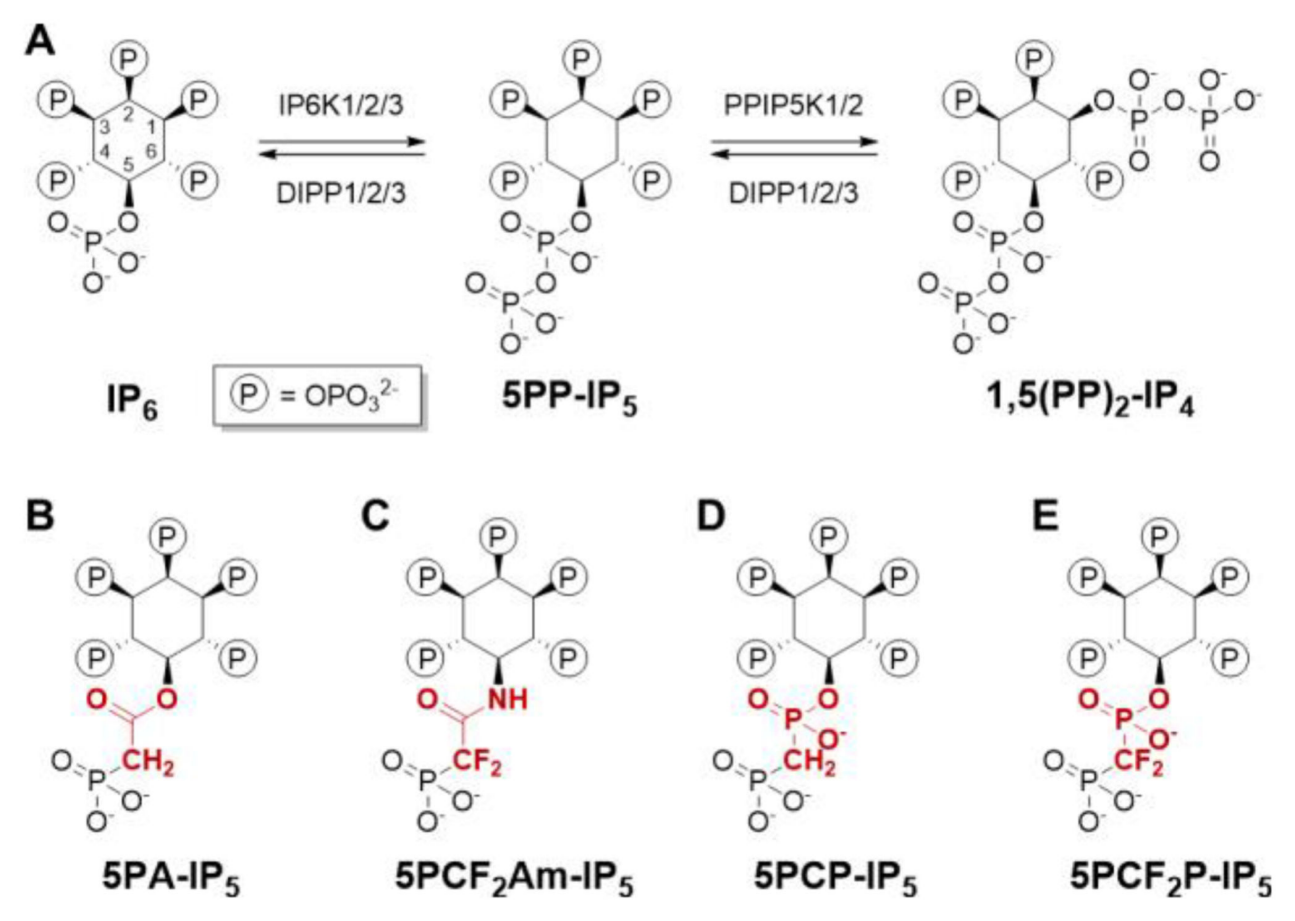
Inositol polyphosphates and their non-hydrolysable analogs. (A) Abbreviated biosynthesis of inositol polyphosphates. (B-E) Structures of metabolically stable analogs of 5PP-IP_5_.

The multitudinous members of the inositol phosphate (IP) family are utilized as cell-signaling molecules by all eukaryotes. A subgroup of IPs known as ‘inositol pyrophosphates’ (PP-IPs; e.g., Figure 1A), which contains chemically and functionally distinct diphosphate groups, is of particular interest. These molecules have been characterized as ‘energetic’,^[1]^ because the hydrolysis of the phosphoanhydride bonds is accompanied by a significant free energy change due to electrostatic and solvation phenomena.^[2]^ These properties underlie the ability of PP-IPs to regulate target protein function by non-enzymatically phosphorylating a pre-existing phosphorylation site on both Ser and Thr.^[3–5]^ The PP-IP diphosphate groups also contribute to ligand specificity through allosteric interactions with protein receptors; prominent mammalian examples include the SPX domain of mammalian XPR1,^[8,9]^ and certain pleckstrin homology domains.^[10–12]^ Separately, the high negative charge density of PP-IPs is also hypothesized to act as an intermolecular electrostatic ‘glue’ to promote formation of functionally significant macromolecular complexes.^[13,14]^ These various mechanisms of action license PP-IPs to regulate an extremely wide range of biological activities.^[14–16]^

Two classes of enzymes synthesize PP-IPs. The IP6Ks install a 5-*ß*-phosphoryl group onto inositol hexakisphosphate (IP_6_),^[17,18]^ yielding 5-diphosphoinositol pentakisphosphate, 5PP-IP_5_, the most abundant inositol pyrophosphate in mammalian cell lines (Figure 1A).^[19]^ PPIP5Ks, in turn, further phosphorylate the 1-position and convert 5PP-IP_5_ to 1,5(PP)2-IP4, also termed IP8 (Figure 1A).^[20,21]^ Reversely, DIPPs (diphosphoinositol polyphosphate phosphohydrolases) can hydrolyze the pyrophosphate moieties to monophosphate groups (Figure1A).^[22,23]^ Understanding PP-IP-protein interactions at the biochemical and the structural level can be stymied by the propensity of the ligand to undergo spontaneous and/or enzymatic hydrolysis. To circumvent these limitations, a series of non-hydrolyzable analogs has been developed (Figure 1B,C,D).^[24–29,6]^ These analogs have been applied as metabolically stable surrogates to investigate PP-IPs function in biochemical assays and structural studies, as well as in cell lysates and inside cells.^[9,30–32]^

The first reported analogs of 5PP-IP_5_ contained either a phosphonoacetate (5PA-IP_5_, Figure 1B) or a methylene bisphosphonate (5PCP-IP_5_, Figure 1D) group in place of the pyrophosphate (PP) moiety.^[24–28]^ In both cases, the oxygen atom of the phosphoanhydride bond was replaced by a methylene group, thereby diminishing hydrolysis. The phosphonoacetate (PA) moiety was readily installed on the inositol ring, and 5PA-IP_5_ bound to the kinase domain of PPIP5K2 (PPIP5K2^KD^) similarly to natural 5PP-IP_5_.^[25]^ In addition, 5PA-IP_5_ enabled the identification of a surface-mounted substrate capture site of PPIP5K2.^[31]^ However, the PA ester can still hydrolyze under highly basic conditions, or in presence of esterases. To address this issue, the Potter group recently developed an α-phosphono-α,α-difluoroacetamide analog, 5PCF_2_Am-IP_5_ (Figure 1C).^[29]^ In this analog, the pyrophosphate mimicking group is bound to the inositol ring *via* an amide bond, which is less prone to hydrolysis than esters. In addition, compared to phosphonates, difluoromethyl phosphonates are considered better bioisosteres of phosphate groups in terms of pK_a_, geometry, and polarity.^[33–36]^ The terminal phosphonate group should thus more closely resemble the *β*-phosphate of 5PP-IP_5_. However, it should be noted that both PA and PCF2Am groups exhibit only two protonation states, instead of three for the natural pyrophosphate. The methylene bisphosphonate (PCP) moiety, on the other hand, has three protonation states and is structurally more similar to pyrophosphate. Although obtaining PCP-IP analogs used to be challenging, the recent use of phosphoramidite chemistry to append the PCP moiety significantly facilitated their synthesis (Figure 1D).^[6]^ Nevertheless, even the PCP moiety might not exactly mirror the protonation state of the pyrophosphate group in biological media. Replacing the central oxygen with the less electronegative methylene moiety will result in an increase of the pKas of the terminal phosphonate group. Therefore, at physiological pH, the analogs will be less strongly ionized than their natural counterparts, which may influence their interactions with proteins. Replacing the methylene group with a difluoromethylene moiety is thus an appealing strategy to improve the properties of these non-hydrolyzable analogs, yet it is anticipated to constitute a significant synthetic challenge.

We now report the synthesis of the difluoromethylene bisphosphonate analog 5PCF_2_P-IP_5_ (Figure 1E), using a fluorinated phosphoramidite reagent. The properties of 5PCF2P-IP5 were then compared systematically to previously described analogs and the natural molecule in different biophysical and biochemical assays. Co-crystallization with the proteins DIPP1 and PPIP5K2 enabled us to scrutinize similarities and disparities of all analogs, as compared to 5PP-IP_5_. The interactions of all analogs with the kinase domain of PPIP5K2 were also examined using ITC and a reverse kinase assay. Finally, we established a chromatographic assay that enabled us to analyze the polarities of all analogs. Taken together, our data provide a benchmark of the advantages and caveats for the use of each analog, and should guide the choice of the appropriate analog(s) for future biological applications.

## Results and Discussion

### Synthesis Of 5PCF_2_P-IP_5_

**Scheme 1:**
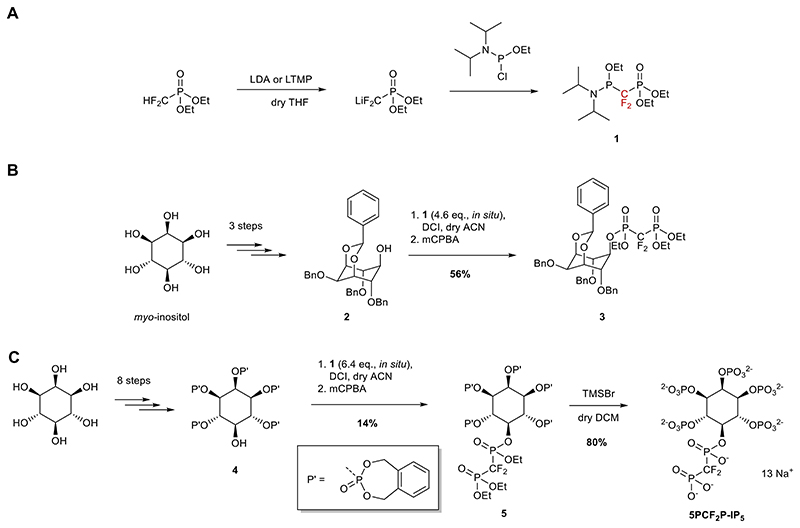
Synthesis of the fluorinated analog 5PCF_2_P-IP_5_. (A) Synthesis of the fluorinated combined phosphoramidite **1** was adapted from a recently reported strategy.^[6,7]^ (B) In the first attempts to obtain 5PCF_2_P-IP_5_, inspired by our previous work on methylene bisphosphonate moieties, the difluoro-bisphosphonate moiety was appended on an early synthesis intermediate^[6]^ (C) In the synthetic route that led to 5PCF_2_P-IP_5_, on the contrary, the difluoro-bisphosphonate moiety should be appended at the very end of the synthesis.

Initial synthetic strategies to obtain PCP-IP analogs employed phosphoryl chloride-based reagents to append the PCP moiety to the inositol scaffold.^[24,27,28]^ This approach required excess of the reagents and resulted in moderate overall yields, which discouraged us to pursue fluorinated versions of these reagents. However, we recently reported improved syntheses for PCP-IP analogs using combined phosphate ester-phosphoramidite reagents.^[6]^ In addition to the high yields obtained with these P^V^-P^III^ reagents, they have the advantage of being modular: they are formed by the reaction of a deprotonated methyl phosphonate derivative with a chlorophosphoramidite, and one can easily alter the protecting groups or derivatize the methyl group. We thus envisioned that replacement of the methyl group by a difluoromethyl group should result in the formation of a “PCF_2_P-amidite” that could subsequently be reacted with appropriately protected inositol derivatives (Scheme 1A).

**Figure 2:**
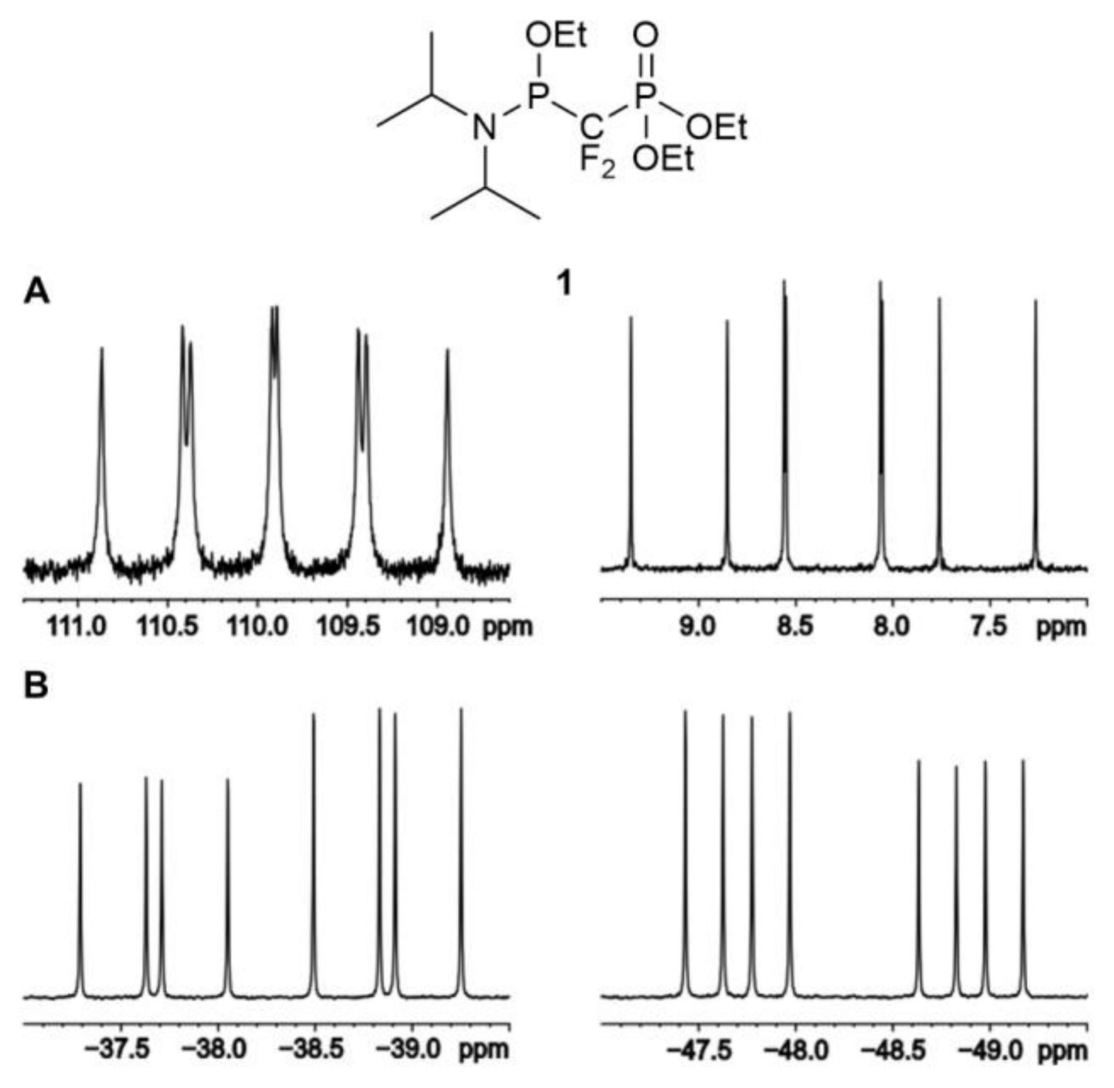
Representative NMR signals of the fluorinated phosphoramidite 1. Selected regions of (A) ^31^P NMR and (B) ^19^F NMR spectra of **1**.

The PCF2P-amidite **1** could be synthesized from commercially available diethyl difluoromethyl phosphonate and *N,N-* diisopropylamine ethyl chlorophosphoramidite, using lithium diisopropylamide (LDA) as a base. It was possible to isolate the product by silica gel chromatography and its formation was confirmed by NMR spectroscopy. The ^31^P NMR spectrum exhibited two signals at 8.3 and 109.9 ppm, corresponding to the P^V^ and P^III^ centers, respectively. Both signals occurred as doublet of doublets of doublets (ddd), reflecting the coupling between the two ^31^P nuclei, as well as coupling to the two diastereotopic ^19^F nuclei (Figure 2). The ^19^F NMR spectrum mirrored the ^31^P spectrum and exhibited two ddd signals at -38.31 and -48.27 ppm, with matching coupling constants (Figure S1).

Attempts to use purified **1** to append the PCF2P moiety to the inositol ring yielded only small amounts of the desired product. Also, increasing the stoichiometric ratio of the PCF2P-amidite did not improve the yields significantly. We thus decided to generate PCF_2_P-amidite **1** *in situ*, and apply it in excess (4.6 eq.), for the reaction with the inositol derivative **2** in the presence of 4,5-dicyanoimidazole (DCI) as an activator,^[37]^ followed by oxidation with mCPBA (Scheme 1B). This strategy yielded the desired coupled product **3** in decent yield (56%, Figure S2). This yield was lower than that obtained with the methylene phosphoramidite, despite a higher stoichiometry of the fluorinated reagent. Attempts to use other activators, such as 5-Ph-tetrazole instead of DCI, did not improve this yield. The fluorinated phosphoramidite is likely more difficult to activate than its methylene counterpart, due to the electron withdrawing effect of the CF_2_ group, which makes the protonation of the nitrogen atom more difficult.

While the coupling yield was still acceptable the major challenge occurred during the subsequent steps. After deprotection of the hydroxyl groups, several side products were observed in which the PCF_2_P moiety had changed position on the inositol ring. A possible explanation is that the electrophilicity of the phosphorus center is enhanced by the by the CF2 group, making it more susceptible to nucleophilic attack by neighboring hydroxyl groups. After various attempts to suppress this phenomenon (see SI for details), we ultimately decided to change the synthetic scheme altogether, and append the PCF2P moiety in the last synthetic step, prior to final TMSBr deprotection: PCF_2_P-amidite **1** was formed *in situ* and was then reacted in excess (6.4 eq.) with protected inositol derivative **4**; a derivative in which protected phosphate groups have already been installed at positions 1,2,3,4 and 6 (Scheme 1C). Coupling was followed by oxidation, to yield the desired product **5**, albeit in low yield (14%). The consistently low yields likely reflect the steric hindrance around the 5-hydroxyl group. Nevertheless, the subsequent final deprotection of **3** in presence of TMSBr proceeded smoothly to provide the desired product, 5PCF_2_P-IP_5_.

### Analysis Of Crystal Complexes Of Dipp1 In Complex With Fluorinated 5PP-IP_5_ Analogs

**Figure 3:**
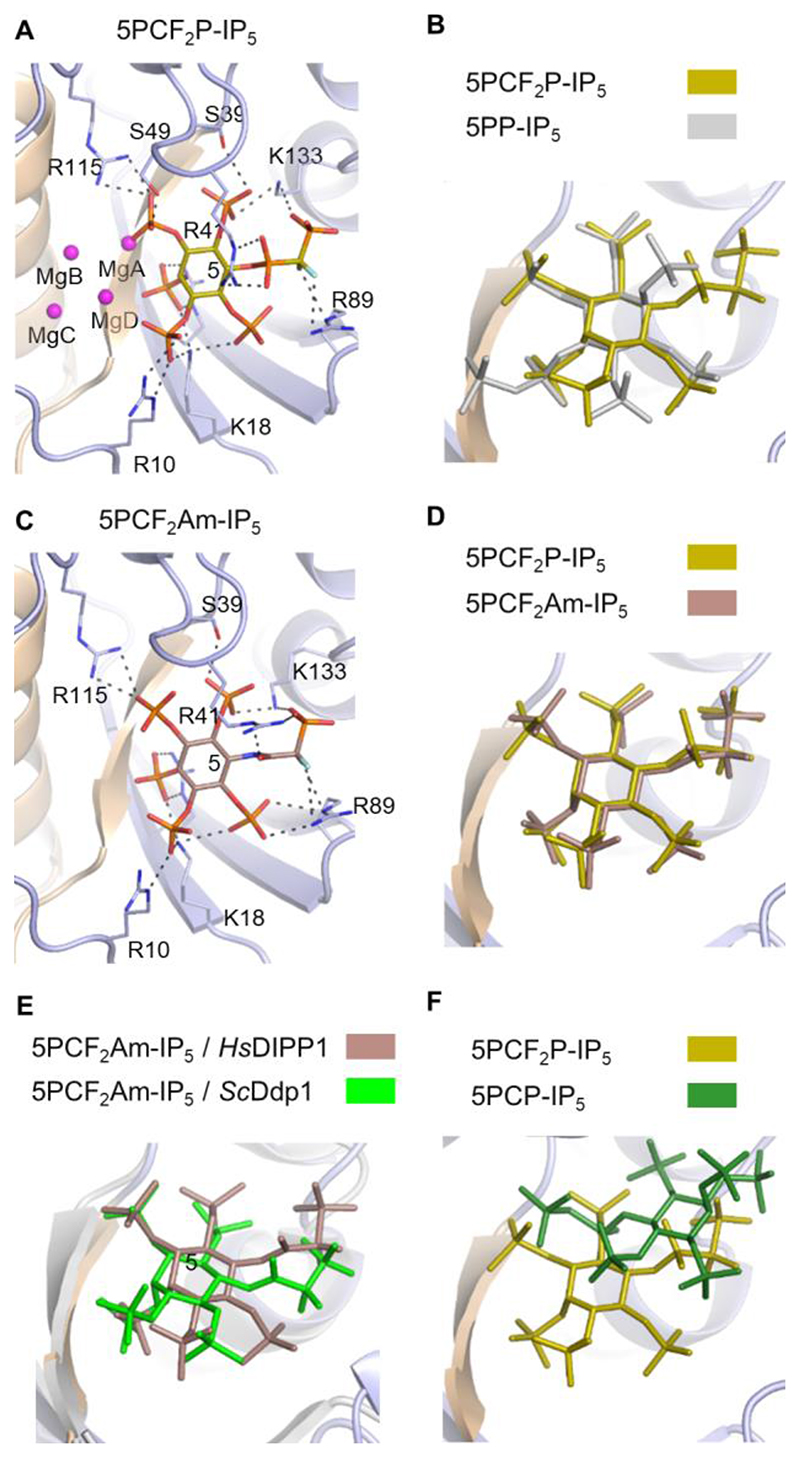
Interactions of 5PCF2P-IP and 5PCF_2_Am-IP_5_ with Dipp1. (A) Stick models are used to depict 5-PCF_2_P-IP_5_ and residues within polar-bond range (<3.2 Å) in a crystal complex with DIPP1 (PDB code: 8G9C); orange denotes phosphorus, red indicates oxygen, blue indicates fluoride, blue denotes nitrogen. The OMIT map is contoured at 2.5 σ and is shown in Figure S4. Four magnesium atoms are depicted as magenta spheres. (B) The orientation of 5PCF_2_P-IP_5_ in panel A (stick model with olive carbons) is superimposed upon 5PP-IP_5_ (dark yellow carbons) in complex with DIPP1, as described in an earlier study ([37]; PDB code, 6WO7). (C) 5PCF_2_Am-IP_5_ (wheat-colored carbons; C-5 is numbered) in a crystal complex with DIPP1 (PDB code: 8G9D). The omit map is contoured at 2.5 σ. (D) Superimposition of 5PCF2P-IP5 in panel A upon 5PCF_2_Am-IP_5_ from panel C. (E, Superimposition of 5PCF_2_Am-IP_5_ from panel C upon 5PCF_2_Am-IP_5_ in a crystal complex with ScDdp1 (light green carbons). (F) Superimposition of 5PCF_2_P-IP_5_ from panel A with 5PCP-IP_5_ in complex with DIPP1 (dark green carbons; [37], PDB code, 6WOG).

To investigate the properties of 5PCF_2_P-IP_5_, we next obtained structural data in complex with the human phosphohydrolase DIPP1. The DIPP1/5PCF_2_P-IP_5_ structure included the four Mg^2+^ atoms we identified in a previous study with the natural substrate 5PP-IP_5_ (Figure 3A).^[38]^ It is interesting that, relative to 5PP-IP_5_, the 5PCF_2_P-IP_5_ is flipped approximately 180° across the C2-C5 axis into a non-productive orientation (i.e., it does not mimic the catalytically-productive positioning of the 5PP-IP_5_ *β*-phosphate; Figure 3B). An almost identical positioning was observed for 5PCF_2_Am-IP_5_ in DIPP1 crystals (Figure 3C,D). These nonproductive ligand presentations also resemble those of 5PCF_2_Am-IP_5_ in a crystal complex with the *S. cerevisiae* Ddp1 (Figure 3E)^[39]^. Perhaps for DIPP1 this particular binding mode is the result of a biologically irrelevant stabilization, promoted by the fortuitous positioning of the fluorine atoms that facilitates polar interactions with R89 (Figure 3A,C). Aliphatic fluorine atoms can form F...H-N hydrogen bonds with nitrogen containing amino acids, in particular arginine, even though these are much less frequent than F...H-C hydrogen bonds. ^[40]^ There are also a few examples in the literature where the interaction between fluorine and an arginine residue in the binding pocket was a possible explanation for the stereoselective binding of CHF phosphonates.^[41]^ Positively charged amino acids such as arginines and lysines are often abundant in protein binding sites of inositol pyrophosphates. Thus, it is possible that a F...H-N hydrogen bond may contribute to a binding mode that is not biologically relevant. It is notable that our previously-described capture of a crystal complex of DIPP1 and 5PCP-IP_5_^[38]^ shows that the 5PCP moiety is oriented towards the active site, but the inositol ring is 5.3 Å displaced relative to that in 5PCF_2_P-IP_5_ (Figure 3F) and overall does not enter the catalytic pocket as deeply.

### Structural Comparison Of 5PP-IP_5_ Analogs In Crystal Complexes With The Kinase Domain Of PPIP5K2

**Figure 4:**
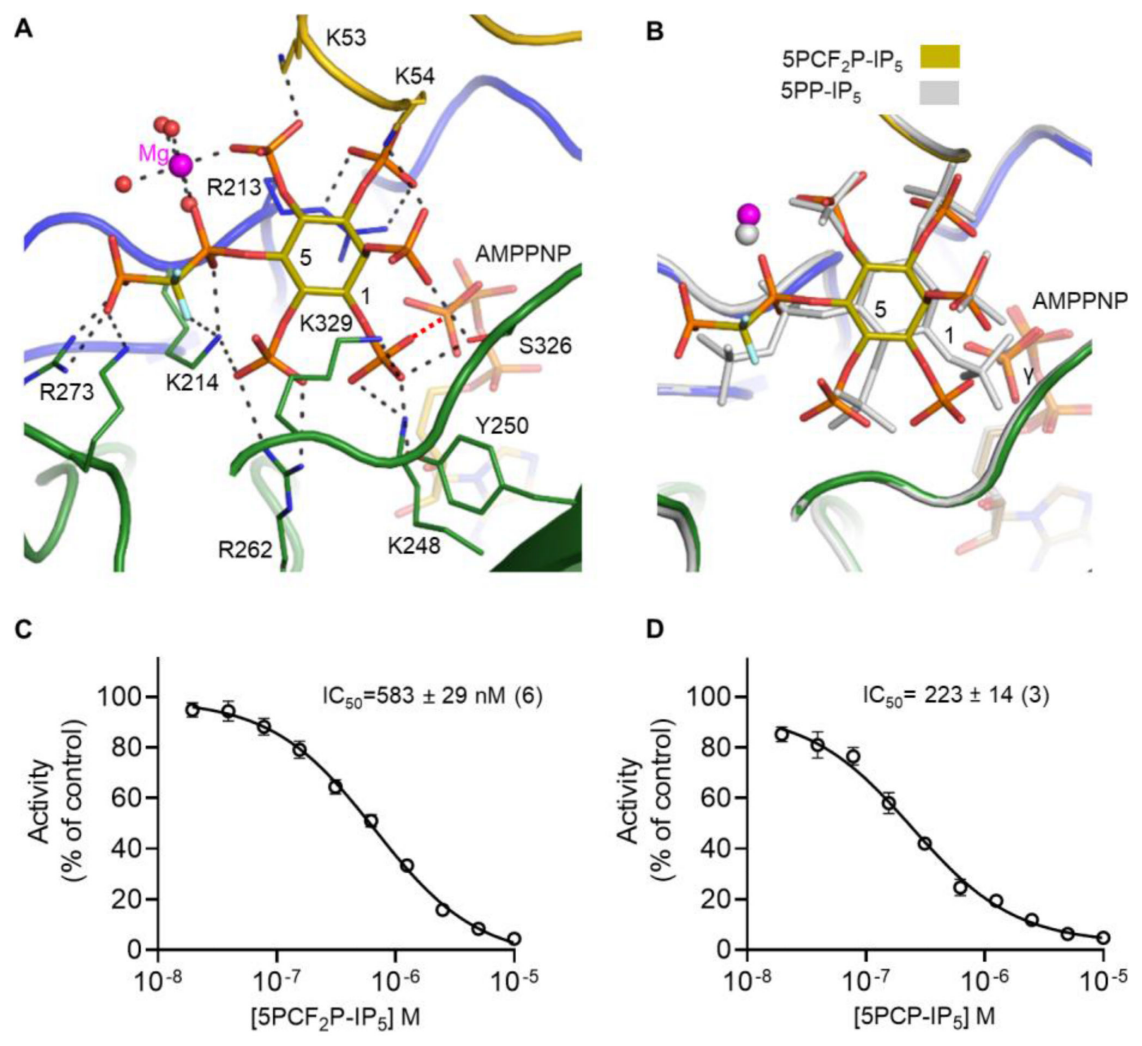
Interactions of 5PCF_2_P-IP_5_, 5PP-IP_5_ and 5PCP-IP_5_ with the PPIP5K^KD^ kinase domain. (A) Structural depiction of the catalytic pocket of the PPIP5K2^41-366^ / 5PCF_2_P-IP_5_ / AMPPNP crystal complex (PDB code: 8G9E). Colour-coded tubes depict protein loops hosting residues that form polar contacts (broken black lines) with PPIP5K2^KD^: yellow for the αβα domain, while blue and green denote different lobes from the ATP-grasp domain (see (39)). 5PCF_2_P-IP_5_ and its interacting residues are depicted as colour-coded stick models: carbons are olive, phosphorus is orange, oxygen is red, nitrogen is blue, and fluorine is cyan, the magenta sphere depicts magnesium, and the red spheres represent water molecules. The OMIT difference map for 5PCF_2_P-IP_5_ is contoured at 5.0 σ and shown in Figure S4. The broken red line depicts the 5.3 Ä distance between the γ-phosphate of AMPPNP and the closest oxygen from the 1-phosphate. (B) Superimposition of PPIP5K2^41-366^ protein complexes containing either 5PP-IP_5_ (PDB = 3T9D; depicted in light gray, 1 - and 5-carbons are labeled) or 5PCF2P-IP5 (color-coded as in panel A). (C, D) IC50 plots for inhibition of the reverse kinase activity of PPIP5K2^1-366^, from a total of 6 (panel C) or 3 (panel D) independent experiments.

Unlike DIPP1, the catalytic site of the PPIP5K2 kinase domain (PPIP5K2^KD^) has not previously been shown to permit multiple ligand binding modes within the active site, for either natural PP-IPs or their analogs.^[25,27,29,31,42,43]^ Consistent with those earlier data, we now describe a 5PCF_2_P-IP_5_ / PPIP5K2^KD^ / AMPPNP crystal complex in which the PP-IP analog is bound in a catalytically productive orientation, in which the presentation of the 1-phosphate is similar to that of the corresponding moiety in 5PP-IP_5_ (Figure 4A,B). The 1-phosphate oxygen atom in PCF2P-IP_5_ is positioned 5.3 Å from the *y*-phosphate of AMPPNP (Figure 4A,B), as compared to 3.4 Å in the 5PP-IP_5_ / PPIP5K2^KD^ / AMPPNP crystal complex.^[42]^ Nevertheless, the placement of the PCF_2_P-IP_5_ 1-phosphate is close enough for a nucleophilic attack distance to posit that this analog might be phosphorylated by the kinase.

There are additional aspects to both the orientation of this analog and several of its polar contacts that closely resemble those of 5PP-IP_5_ (Figure 4A,B). Specifically, Arg273 forms two polar contacts (< 3.2 A) with the 5-*ß*-phosphate of PCF_2_P-IP_5_, while Lys214 coordinates the adjacent 6-phosphate (compare Figure 4A with data in reference ^[42]^). Another conserved feature of the 5PCF_2_P-IP_5_ / PPIP5K2^KD^ complex is the capture of a magnesium ion through a network of polar interactions with four water molecules, the 5-*α*-phosphate, and the 4-phosphate (Figure 4A,B). It is notable that the 5PCP-IP_5_ analog also recruits a corresponding Mg^2+^ ion into the crystal complex with PPIP5K2^KD,[27]^ whereas Mg^2+^ is missing from the PPIP5K2^KD^ complexes with either 5PCF_2_Am-IP_5_^[29]^ or 5PA-IP_5_.^[31]^ The nature of certain polar contacts with PPIP5K2^KD^ are unique to the composition of the 5PCF_2_P moiety. Most notably, 5PCF_2_P-IP5 forms three polar interactions with Lys214, two of them are with fluorine atoms and the third is with the α-phosphate oxygen (Figure 4A). With 5PP-IP_5_ as the ligand, Lys214 forms two polar contacts with the bridging oxygen and a third contact with one of the 5-*ß*-phosphate oxygens.^[42]^

An alternative method to study the interaction of PP-IP_5_ analogs with PPIP5K2^KD^ is to derive IC_50_ values for inhibition of a “reverse-kinase” assay that records ATP formation when the enzyme is incubated with ADP and 1,5(PP)_2_-IP_4_.^[29,31]^ This approach avoids the need to use radiolabeled material and HPLC analysis. Moreover, the assay of ATP production is inherently more sensitive compared to measuring ATP consumption. Using this approach we found that 5PCF_2_P-IP_5_ inhibited ATP production (i.e., 1,5(PP)_2_-IP_4_ dephosphorylation) (Figure 4C and Table 1), albeit with slightly lower potency (IC50 = 583 nM) than published IC_50_ values for 5PA-IP_5_ (129 nM)^[31]^ and 5PCF_2_Am-IP_5_ (375 nM)^[29]^ The IC50 value for 5PCP-IP_5_ in this assay amounted to 223 nM (Figure 4D). In addition to the catalytic pocket, the PPIP5K2 kinase reaction cycle also utilizes a surface-mounted substrate capture site; the latter was initially identified through its binding of 5PA-IP_5_.^[31]^ In contrast, the electron density for 5PCF_2_P-IP_5_ was only observed within the catalytic pocket (Figure 4A), as is also the case for crystal complexes of PPIP5K2^KD^ with either 5PCF_2_Am-IP_5_^[29]^ or 5PCP-IP_5_.^[27]^ Such data underscore the biological value of specific chemical properties of individual analogs. In this case, the higher affinity of the capture site for less polar molecules may explain why 5PA-IP_5_ is the only analog so far detected in this site.

### Biophysical And Chemical Comparison Of The Interactions Of 5PP-IP_5_ Analogs With PPIP5K2^KD^

**Figure 5:**
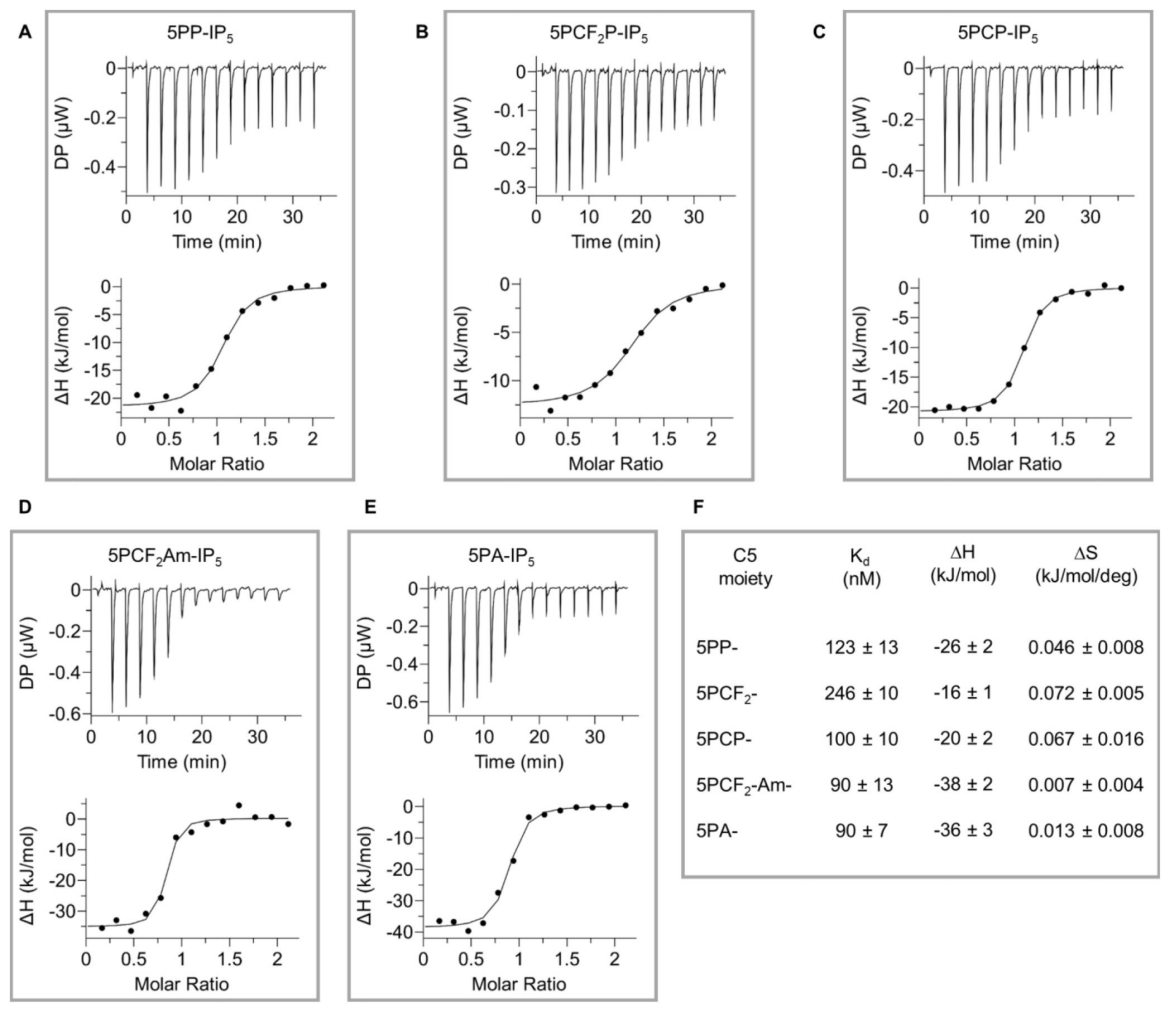
Analysis of ligand binding to the PPIP5K2^KD^ by ITC. Test solutions contained 7.5 μM of PPIP5K2^1-366^ in the sample cell and 75 μM ligand in the syringe. Other details are provided in the Methods Section. Representative thermograms (1 of 3 replicates) are shown for titration of either (A) 5PP-IP_5_; (B) 5PCPF_2_P-IP_5_; (C) 5PCP-IP_5_; (D) 5PCF_2_Am-IP_5_; (E) 5PA-IP_5_. (F) Corresponding thermodynamic data.

We next used isothermal titration calorimetry (ITC) to analyze the interactions of PPIP5K2^KD^ with 5PP-IP_5_ and each of the four analogs studied herein (Figure 5A-E). The apparent Kd for the natural substrate was 123 nM; the K_d_ values for the analogs were in the range from 90 nM to 246 nM (Figure 5F). Interestingly, the entropic components of the binding energies for 5PCF_2_Am-IP_5_ and 5PA-IP_5_ were both relatively low, perhaps reflecting the more rigid nature of their C5 moieties. At the same time, the enthalpic gain for binding was largest for 5PCF_2_Am-IP_5_ and 5PA-IP_5_. A possible explanation may be that these two ligands are less strongly hydrated, due to their lower overall charge. The enthalpic cost for dehydration of 5PCF_2_Am-IP_5_ and 5PA-IP_5_ is therefore lower, compared to the other molecules, resulting in a higher enthalpic gain overall. A less strongly hydrated ligand will also release fewer water molecules upon binding, consistent with the lower entropic gains.

Six-membered carbon rings have an inherent conformational flexibility. While the staggered chair conformer of PP-IP_5_ is mimicked by each of the analogs, more subtle puckering away from this idealized structure could be induced by substituents and any accompanying changes in intramolecular hydrogen bonding.^[44]^ Such phenomena are hard to discern from the data in Figure 4, in which the orientations of the inositol rings are also influenced by adjustments in protein conformation. However, more direct ring-on-ring superimpositions indicated that none of the analog’s substituents have a detectable impact upon ring puckering (Figure S3).

Lastly, we also developed a protocol by which we can infer the relative polarities of each of the analogs. This strategy emerged from our previous demonstration that PPIP5K2^KD^ has a finite kinase activity towards 5PA-IP_5_ and the product can be detected (but not accurately quantified) by PAGE analysis of reactions containing exceptionally high concentrations of enzyme and substrate.^[25]^ As shown above, 5PA-IP_5_ and the other analogs each bind in potentially productive orientations in which the 1-phosphate is presented to the active site. Therefore, we attempted to radiolabel each of the analogs by incorporation of ^[33]^Pi from ^[33]^P-γ-ATP, with the objective of resolving each of the ^[33]^P-labeled products by anion-exchange HPLC. We reasoned that this assay could provide an empirical comparison of the relative degree of acidity of each reaction product - and more instructively - the substrates themselves. It should be emphasized that this experiment cannot inform on the relative rates of phosphorylation, in large part because the experimental protocol (low mass levels of high-specific radioactivity of ^[33]^P-γ-ATP) are not appropriate for biologically-relevant kinetic determinations.

**Figure 6:**
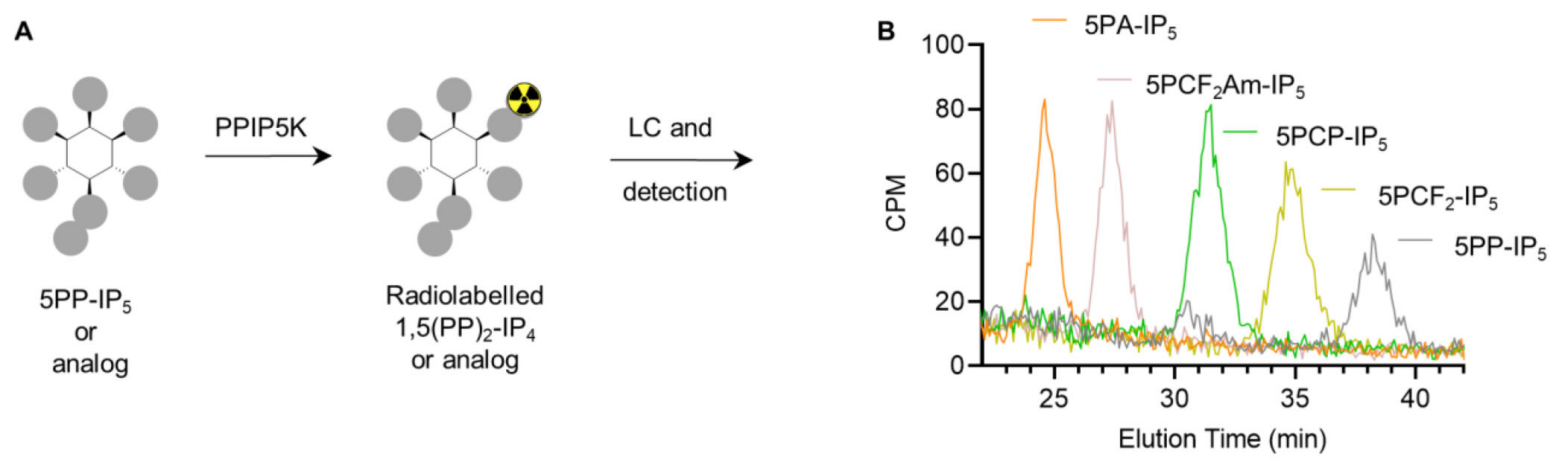
HPLC analysis of the products of PPIP5K-mediated phosphorylation of 5PP-IP_5_ and the various analogs. (A) Principle of the assay. PPIP5K2^1-366^ was incubated with [^33^P-γ]ATP and either 5PP-IP_5_ or one of the analogs (each of which is color-coded as described in the figure), as described in Experimental Section. The [^33^P]-labeled reaction products were resolved in individual HPLC runs, and the elution data are incorporated into a single plot. (B) A representative data set is shown, from a total of 3 independent experiments.

The results of these experiments are shown in Figure 6. The relative HPLC elution times for the products of each of the tested substrates (earliest to latest) is as follows: 5PA-IP_5_ < 5PCF_2_Am-IP_5_ < 5PCP-IP_5_ < 5PCF_2_P-IP_5_, followed lastly by the natural substrate, 5PP-IP_5_. These observations suggest that the new 5PCF_2_P-IP_5_ analog most closely resembles the natural molecule with regards to its protonation state.

## Conclusion

We have developed a synthesis of 5PCF_2_P-IP_5_, an analog of 5PP-IP_5_ with a metabolically stable difluoromethylene bisphosphonate, and have compared its properties with other reported analogs. Difluoromethylene phosphonate is considered a better bioisostere of phosphate than methylene phosphonate,^[33–35]^ yet the synthesis of difluoromethylene bisphosphonate analogs of diphosphorylated biomolecules is a challenge.^[34]^ Building upon the success of the “combined phosphoramidite” approach to append methylene bisphosphonate moieties to biomolecules,^[6,7]^ we were able to obtain the desired 5PCF_2_P-IP_5_, albeit in modest yields.

We then sought to compare the properties of this new analog with those of natural 5PP-IP_5_, but also of other reported structurally closely-related analogs. To do so, we utilized human DIPP1 and the kinase domain of PPIP5K2 (PPIP5K2^KD^), each of which has its own analytical advantages. The DIPP1 phosphatase has a flexible ligand binding domain whereas the PPIP5K kinase domain displays higher ligand specificity. In the constrained environment of the PPIP5K kinase domain 5PCF_2_P-IP_5_ reproduced the binding orientation of 5PP-IP_5_, similar to other analogs. In the more flexible DIPP it was striking to see that both fluorinated analogs (5PCF_2_P- and 5PCF_2_-Am) were flipped 180° when compared to 5PP-IP_5_ and the non-fluorinated analogs 5PA- and 5PCP-IP_5_. Although the fluorinated analogs have protonation states that are closer to the natural molecule at physiological pH, the introduction of the fluorine atoms leads to the formation of a polar interaction with a nearby arginine residue, favouring the flipped orientation. Given the high density of positively charged residues in PP-IP binding sites, it seems likely that such polar interactions between fluorine and neighboring lysine or arginine side chains can contribute to the binding orientation and binding strength of the fluorinated analogs.

We also examined the interactions of analogs with PPIP5K^KD^ from a biochemical perspective. All analogs inhibited its kinase activity, within the 0.1-1 μM range. It is interesting to note that fluorinated analogs have higher IC_50_ values than the non-fluorinated ones. In ITC experiments with PPIP5K2^KD^ all analogs have Kd values in the 90 to 123 nM range, except for the 5PCF2P-analog (246 nM). The PA and PCF_2_Am analogs exhibited significantly lower binding entropy than 5PP-IP_5_ and the other analogs. Finally, an HPLC assay enabled us to compare indirectly analogs by global polarity, showing that the 5PCF_2_P-analog was the closest to 5PP-IP_5_, followed by 5PCP-IP_5_ and the less charged 5PCF_2_Am- and PA-analogs.

Taken together, our data show that none of the analogs perfectly captures all of the features of natural 5PP-IP_5_, but that the accuracy with which they reproduce the properties of 5PP-IP_5_ depends upon the nature of the interaction and the assay. Therefore, the choice of an analog to answer a biological question should take into account the physicochemical features highlighted here, the nature of the studied interaction, and the synthetic access to the desired analog.

## Experimental Section

### Synthesis and characterization

General information, detailed procedures, complete characterization and NMR spectra are provided in the electronic Supporting Information.

#### Compound 1

The synthesis of compound **1** was adapted from published procedures.^[6,7,45]^ 2,2,6,6-tetramethylpiperidine (178 μL, 1.05 mmol) was diluted in dry THF (3mL) under a dry N_2_ atmosphere. The solution was cooled to 0°C (ice bath) and a 2.5M solution of BuLi in hexanes (0.42 mL, 1.05 mmol) was added dropwise. The solution turned yellow, and was stirred for 45 min at 0°C. It was then cooled in a CO_2_ (s)-acetone bath. Diethyl (difluoromethyl) phosphonate (0.16 mL, 1.02 mmol) was added dropwise, and reaction mixture (RM) was stirred at -78°C for an hour. A solution of diethyl chlorophosphoramidite (231.6 mg, 1.09 mmol) was prepared in dry hexane (0.3 mL), filtered over a 0.45μm PTFE syringe filter and quickly added to RM at -78°C. RM was stirred for 1h30 at -78°C. Absolute EtOH (0.1 mL) was added, and RM was poured into a mixture of saturated aqueous NaHCO_3_ (15 mL) and DCM (15 mL). After a quick extraction, the organic layer was dried over Na2SO4, filtered and concentrated to a yellow-orange oil. The crude material was purified by silica gel column chromatography (high-purity silica) (10% EA in hexane). A small amount of the desired product was obtained as a slightly yellow oil (11 mg, 0.030 mmol, 3 %).

#### Compound 3

Anhydrous THF (4 mL) was introduced in an oven-dried 50-mL RBF under a dry N_2_ atmosphere, followed by diisopropylamine (147.6 μL, 1.05 mmol). The solution was cooled in a CO_2_(s)-acetone bath and degassed for 5 min under high vacuum before being restored to a dry N_2_ atmosphere. A 2.5 M BuLi solution in hexanes (0.42 mL, 1.05 mmol) was added dropwise, and the reaction mixture was stirred at -78°C for 30 min. A solution of diethyl difluoromethylphosphonate (156.8 μL, 1.0 mmol) in dry THF (1 mL) was added dropwise. The resulting solution was stirred for 45-50 min at -78°C. A 0.9°M solution of diethylchlorophosphoramidite was prepared in dry hexane and filtered over a 0.45 μm PTFE syring filter, before 1.1 mL of this solution (1 mmol) were quickly added to the reaction mixture. After 2 h, the cooling bath was removed and solvents were removed by rotary evaporation. The resulting orange-brown crude was dried under high vacuum (15 min), then put under a dry N_2_ atmosphere. It was redissolved in 1 mL dry ACN and cooled in an ice-brine bath. A solution of Ins derivative **2** (116 mg, 0.215 mmol) in dry acetonitrile (1.5 mL) was added at 0°C, followed by DCI (224 mg, 1.9 mmol). RM was stirred overnight, over which time it reached RT. A solution of mCPBA (340 mg, 1.52 mmol) in dry ACN (1 mL) was added dropwise at -20°C, and RM was stirred for 1h30 min at -20°C then 30 min at RT. RM was diluted with sat. aq. Na_2_S_2_O_3_ (30 mL) and extracted with EA (30 mL). the organic layer was then washed with sat. aq. NaHCO_3_ (30 mL) and brine (30 mL). The organic layer was dried over Na2SO4, filtered and evaporated. It was purified by silica gel flash column chromatography (25 to 50% EA in hexane), to yield the desired compound as a white solid (98.6 mg, 0.121mmol, 56%).

#### Compound 5

Diisopropylamine (147 μL, 1.05 mmol) was dissolved in 4 mL dry THF under a dry N2 atmosphere. The solution was cooled in a CO_2_(s)-acetone bath, and degassed under high vacuum, before being restored to a dry N_2_ atmosphere. A 2.5 M BuLi solution in hexanes (0.42 mL, 1.05 mmol) was added dropwise, and the reaction mixture was stirred at -78°C for 30 min. A solution of diethyl difluoromethylphosphonate (156.8 μL, 1.0 mmol) in dry THF (1 mL) was added dropwise. The resulting pale yellow solution was stirred for 40-50 min at -78°C. A 1°M solution of diethylchlorophosphoramidite was prepared in dry hexane and filtered, before 1 mL of this solution (1 mmol) added quickly to reaction mixture. After 2 h, the cooling bath was removed and solvents were removed under reduced pressure. The resulting orange-brown crude was dried under high vacuum (15 min), then put under a dry N_2_ atmosphere. A solution of Ins derivative (170 mg, 0.156 mmol) in dry acetonitrile (3 mL) was added at 0°C, followed by DCI (336 mg, 2.8 mmol). RM was stirred overnight at 0°C to RT. mCPBA (334 mg, 1.5 mmol) was added portionwise at 0°C, and RM was stirred for 20-30 min at 0°C then 15-20 min at RT. RM was diluted with EA (25 mL) and washed with sat. aq. Na_2_S_2_O_3_ (20 mL), sat. aq. NaHCO_3_ (20 mL) and brine (20 mL). The organic layer was dried over Na2SO4, filtered and evaporated. It was purified by silica gel flash column chromatography (0 to 10% MeOH in DCM), then by preparative HPLC to yield the desired compound as a white solid (28 mg, 0.021mmol, 14%).

#### Preparative HPLC conditions

XBridge. A = water, no TFA. B = ACN, no TFA. 30 mL/min. 2 min 50%B, then 50 to 70%B in 4 min, then 70%B for 2 min. detection 214 nm. Product @ 4.4 min, starting material @ 3.6 min).

*5PCF_2_P-IP_5_.* Compound **5** (19 mg, 14 μmol) was dissolved in dry DCM (750 μL) under a N_2_ atmosphere. TMSBr (250 μL) was added at 0°C, and the reaction mixture was left to reach RT and to stir for 6 h. Solvents were removed by rotary evaporation, and 3 mL MeOH were added to the resulting residue. Solvent was again removed by rotary evaporation. The crude residue was taken up in Et2O (3 mL) and 1M TEAB (3 mL). The aqueous layer was washed once more with Et2O (3 mL), then coevaporated several times with MeOH, and lyophilized The resulting crude was purified by anion exchange chromatography on a 20 mL Q H column (A: water, B: 1M NH_4_HCO_3_ pH 7.6). Fractions containing the desired compound were lyophilized and pooled. The resulting white solid was finally redissolved in water and stirred overnight with a Chelex resin. The flowthrough was lyophilized, yielding 11 mg of the desired compound as a white solid.

### Biochemical assays

Protein purification and crystallization procedures are detailed in the electronic Supporting Information.

#### PPIP5K2KD reverse kinase assay

For the reverse kinase assay, PPIP5K2^1-366^ (2.5 μg mL^-1^) was incubated at 25 °C for 30 min with 20 μL buffer containing 20 mM Tris-HCl, pH 7.5, 10 mM MgCl_2_, 0.1 mM ADP, 100 nM [1,5]PP-IP_4_ and various concentrations of either 5PCP-IP_5_ or 5PCF_2_P-IP_5_. The generated ATP was measured using a Molecular Probes ATP Determination kit (Thermo Fisher Scientific catalog number A22066). The IC50 value was calculated using GraphPad Prism.

#### Isothermal titration calorimetry

Calorimetry experiments were performed using a MicroCal PEAQ-ITC (Malvern Panalytical) with 7.5 μM recombinant PPIP5K21-366 in the sample cell and 75 μM of ligand in the syringe, each of which was maintained at 25°C in buffer containing 20 mM HEPES, pH 7.2, 150 mM KCl, 0.05 mM EDTA, 1mM AMP-PNP, 1mM MgCl_2_ as indicated. The sample cell (volume = 204 μL) and the syringe were cleaned before each run. Thermograms were constructed from 13 injections, each of which involved 3 μL of ligand delivered for 6 s, with an equilibration time of 150-300 s between each injection. The stirring speed was set to 750 rpm. Data were fitted to a single binding site model using the analysis software provided by the manufacturer. At least three runs were performed for each condition.

#### HPLC Analysis

Kinase activity was studied at 37°C by incubating 700 ng PPIP5K2^1-366^ separately with each individual 10 μM test compound and 50 μM ATP plus 60,000 CPM of [^33^P-γ]ATP for 30 mins. Assays were quenched with 0.2 volumes of 2 M perchloric acid, neutralized, and [^33^P]-labeled products were analyzed by ion-exchange HPLC, using a 4.6 × 125 mm, 5 μm PartiSphere SAX column. The elution gradient (1 ml/min) was generated by mixing Buffer A (1 mM Na2EDTA) with Buffer B (Buffer A plus 2.5M NH_4_H_2_PO_4_, pH 4.0); the elute was mixed with 2.5 mL/min Monoflow4 scintillation liquid (National Diagnostics) and radioactivity was monitored with an in-line counter.

## Supplementary Material

Supplementary Information

## Figures and Tables

**Table 1:** Inhibition of 5PP-IP_5_ hydrolysis by PPIP5K2KD

Compound	IC50	Reference
5PCF_2_P-IP_5_	583 nM	This work
5PCP-IP_5_	223 nM	This work
5PCF_2_Am-IP_5_	375 nM	[29]
5PA-IP_5_	129 nM	[31]
