# Supplementary material for: Fluorination Influences the Bioisostery of Myo‐Inositol Pyrophosphate Analogs
Source: Chemistry. 2023 Oct 19;29(67):e202302426. doi: 10.1002/chem.202302426 (PMC7615343; doi:10.1002/chem.202302426)

Chemistry–A European Journal

Supporting Information

Fluorination Influences the Bioisostery of *Myo*-Inositol Pyrophosphate Analogs

Sarah Hostachy, Huanchen Wang, Guangning Zong, Katy Franke, Andrew M. Riley, Peter Schmieder, Barry V. L. Potter, Stephen B. Shears,* and Dorothea Fiedler*

Table of Contents

Supporting figures, table and scheme 2
General Information 8
Abbreviations 8
Solvents and chemicals 8
Silica gel column chromatography 8
NMR 8
HPLC 8
Reported compounds 8
Compound 1 9
Compound 3 10
Compound 5 11
Compound 5PCF$_2$P-IP$_5$ 12
Biochemical assays 13
Protein expression and purification. 13
Crystallization and structure determination 13
PPIP5K2^{KD} reverse kinase assay 13
Isothermal titration calorimetry 13
HPLC Analysis 13
Author Contributions 14
References 14
Appendices: NMR spectra 14
^{1}H NMR spectrum of compound 1 15
^{19}F NMR spectrum of compound 1 15
^{31}P NMR spectrum of compound 1 16
^{1}H NMR spectrum of compound 3 17
^{13}C NMR spectrum of compound 3 17
^{19}F NMR spectrum of compound 3 18
^{31}P NMR spectrum of compound 3 18
^{1}H NMR spectrum of compound 5 19
^{13}C NMR of compound 5 19
^{19}F NMR spectrum of compound 5 20
^{31}P NMR spectrum of compound 5 20
^{1}H NMR spectrum of 5PCF$_2$P-IP$_5$ 21
^{19}F NMR spectrum of 5PCF$_2$P-IP$_5$ 21
^{31}P NMR spectrum of 5PCF$_2$P-IP$_5$ 22

Supporting figures, table and scheme

Table S1. Data collection and refinement statistics.

PDB Accession IDs	8G9C	8G9D	8G9E
Protein	DIPP1	DIPP1	PPIP5K2
Ligand	5PCF$_2$P-IP$_5$	5PCF$_2$Am-IP$_5$	5PCF$_2$P-IP$_5$
Data Collection			
Space group	P2$_1$2$_1$2$_1$	P2$_1$2$_1$2$_1$	P2$_1$2$_1$2$_1$
Cell parameters a, b, c (Å)	46.7,<br>59.6,<br>62.4	46.4,<br>59.7,<br>62.4	88.1<br>109.9<br>41.1
Resolution (Å) *	50.0-1.40 (1.42)	50.0-1.60 (1.63)	38.5-1.75 (1.78)
R_{merge}*	0.057 (0.549)	0.036 (0.317)	0.082(0.672)
I/σ *	51.3 (3.6)	19.7 (3.1)	13.7(2.2)
Completeness (%)*	92.7(90.1)	98.9 (99.9)	95.0(98.0)
Redundancy*	13.7 (11.4)	6.0 (5.0)	3.6 (3.5)
Refinement			
Resolution (Å)*	1.4 (1.43)	1.6 (1.64)	1.75(1.79)
No. reflections	30984	20338	40319
R_{work}*	17.0 (25.1)	19.3 (22.6)	12.4 (17.3)
R_{free}*	18.8 (25.0)	22.5 (29.0)	17.5 (22.9)
No. atoms			
Protein	1134	1147	2565
Ligand/ion	*49*	*41*	*78*
Solvent	111	133	369
B-factors (Å^2)			
Protein	20.5	16.2	24.1
Ligand/ion	33.0	27.7	23.2
Solvent	33.3	28.0	37.1
R.m.s. deviations			
Bond length (Å)	0.017	0.014	0.01
Bond angle (°)	2.30	2.12	1.62

*The numbers in parentheses are given for the highest-resolution shell

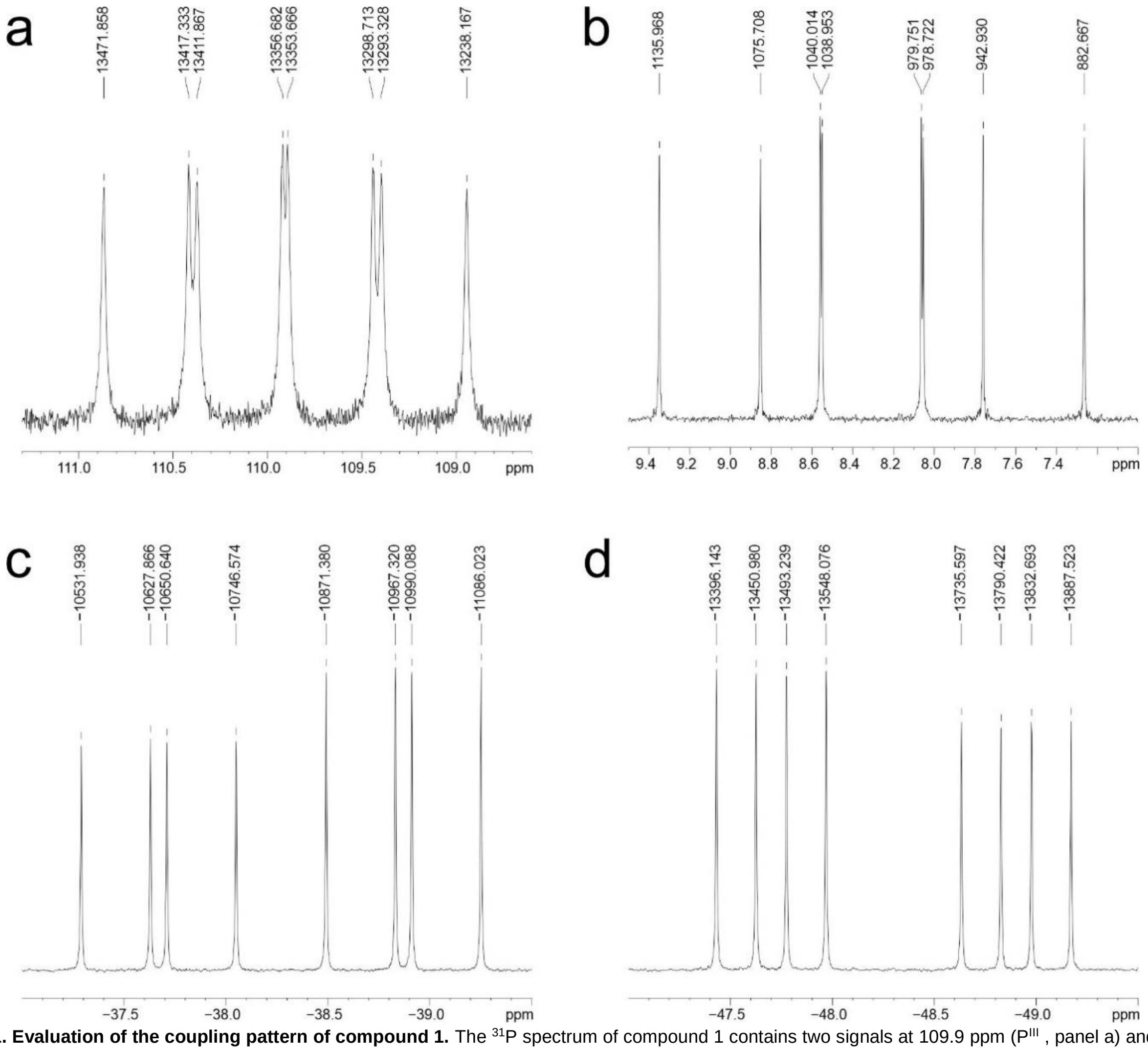

Figure S1. Evaluation of the coupling pattern of compound 1. The ^{31}P spectrum of compound 1 contains two signals at 109.9 ppm (P^{III} , panel a) and 8.3 ppm (P^{V}, panel b). Both signals occur as doublet of doublets of doublets (ddd). The ^{2}J(PP) coupling is 60.3 Hz. Both ^{31}P nuclei show two different ^{2}J(PF) couplings to the two diasterotopic ^{19}F nuclei. The ^{2}J(PF) couplings of the P^{III} nucleus are 54.8 Hz and 118.7 Hz resulting in a clear ddd pattern. The ^{2}J(PF) couplings of the P^{V} nucleus are 97.1 Hz and 95.9 Hz, the similarity of the two coupling constants give the multiplet the appearance of a dt multiplet if the spectrum is processed with line broadening. Without line broadening the ddd pattern is easily evaluated.The same couplings are also visible in the ^{19}F spectrum, since the lowfield ^{19}F (panel c) shows two ^{2}J(PF) couplings of 95.9 Hz and 118.7 Hz and the highfield ^{19}F (Figure d) two ^{2}J(PF) couplings of 54.8 Hz and 97.1 Hz, the ddd pattern are clearly visible. Since the ^{2}J(FF) coupling has a size of 339.5 Hz which is in the range of the difference of the chemical shift (approx. 10 ppm / 2820 Hz), an AB_q pattern is visible. Full evaluation of the pattern results in a minor correction of the chemical shift, the values are -48.27 ppm and -38.31 ppm.

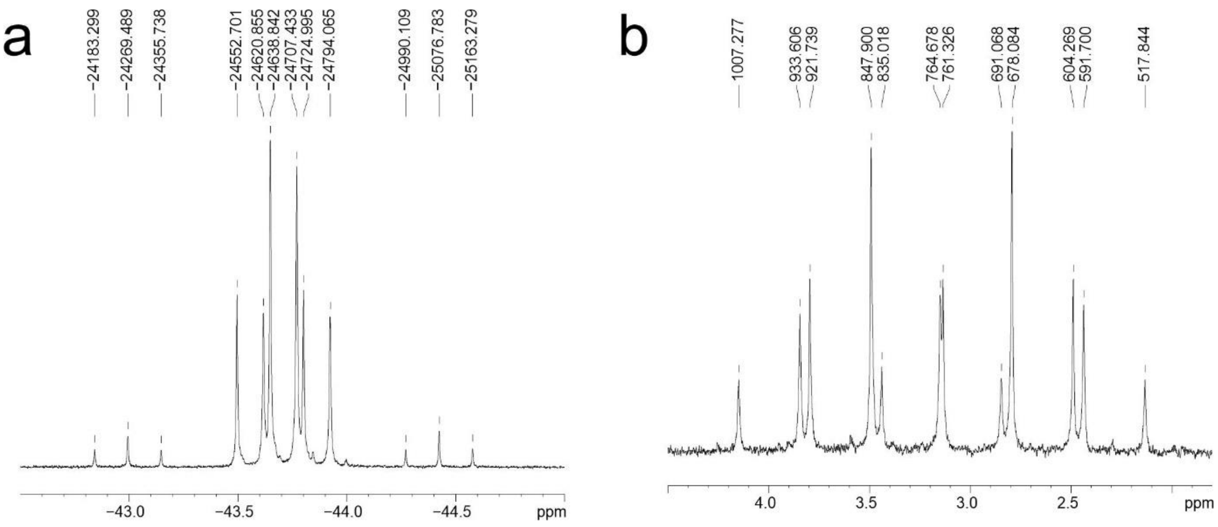

Figure S2. Evaluation of the coupling pattern of compound 3. A detailed evaluation of the ^{31}P and ^{19}F spectra was performed with spectra recorded at 600 MHz (^{1}H frequency). Since this reduces the "roofing" the spectra were easier to analyze. Both the ^{19}F spectrum (panel a) and the ^{31}P spectrum (panel b) of compound **3** show two signals which are close together in frequency. Since the ^{2}J(FF) coupling (389.3 Hz) as well as the ^{2}J(PP) coupling (73.8 Hz) are in the range of the difference of the chemical shifts of the respective nuclei, both spectra show an AB_q pattern. The ^{2}J(PF) couplings between the ^{19}F and the ^{31}P nuclei are all of the same size (86.3 Hz), therefore a triplet is superimposed onto every peak of the AB_q pattern (which is a doublet of doublets). The spectra therefore show a doublet of doublets of triplets. Full evaluation of the AB_q pattern yield ^{31}P chemical shifts of 2.66 ppm and 3.62 ppm and ^{19}F chemical shifts of - 43.92 ppm and - 43.50 ppm.

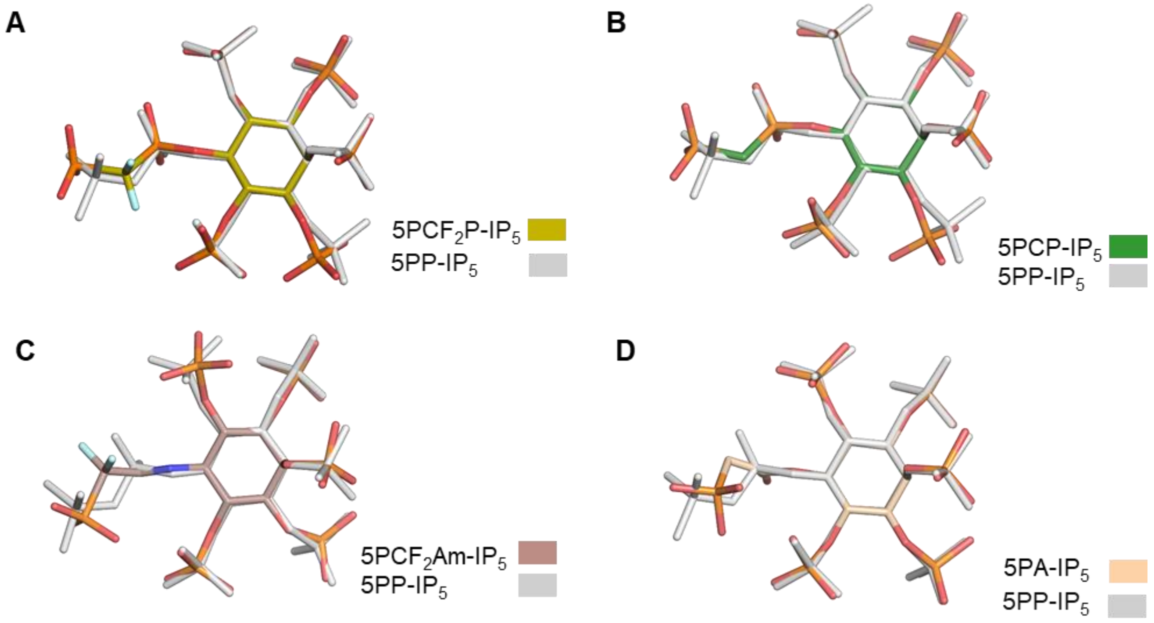

Figure S3. Structural properties of 5PP-IP$_5$ and the various analogs. Using the inositol ring as a reference point, we performed RMSD-minimized alignments each of each analogue upon 5PP-IP$_5$, without reference to the protein. All ligands are shown as stick models, 5PP-IP$_5$ is uniformly colored gray, whereas the analogs are highlighted as follows: phosphorous is orange, oxygen is red, nitrogen is blue, and the carbons are color coded as indicated by the keys in each panel. (A) 5PCPF$_2$P-IP$_5$; (B) 5PCP-IP$_5$; (C) 5PCF$_2$Am-IP$_5$; (D) 5PA-IP$_5$. The data for the structure of 5PCPF$_2$P-IP5 arise from the current study. PDB accession codes for the other ligands are as follows: 3T9D for 5PP-IP$_5$, 5DGH for 5PCP-IP$_5$, 6N5C for 5PCF$_2$Am-IP$_5$, and 4HN2 for 5PA-IP$_5$.

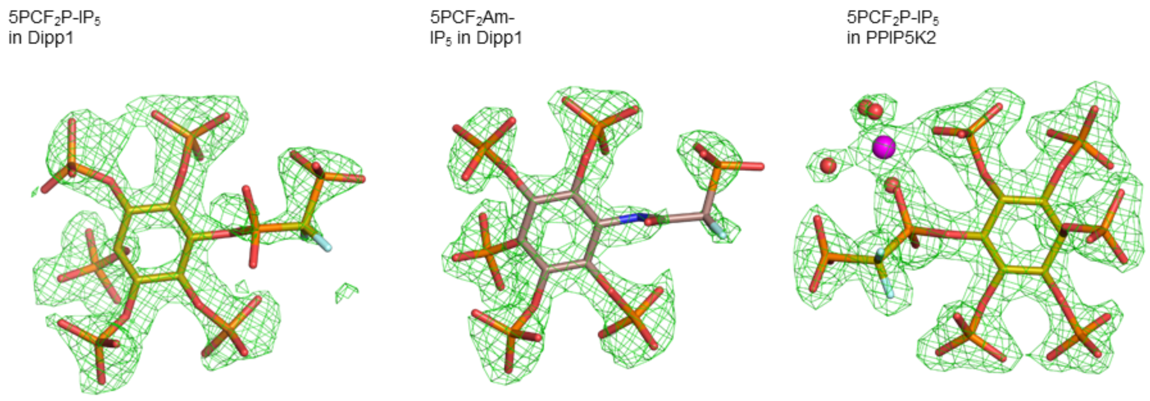

Figure S4. The OMIT difference maps for 5PCF$_2$P-IP$_5$ and 5PCF$_2$Am-IP$_5$ in Dipp1 are contoured at 2.5 σ. The OMIT difference map for 5PCF$_2$P-IP$_5$, Mg atoms, and surrounding water molecules in PPIP5K2 is contoured at 5.0 σ.

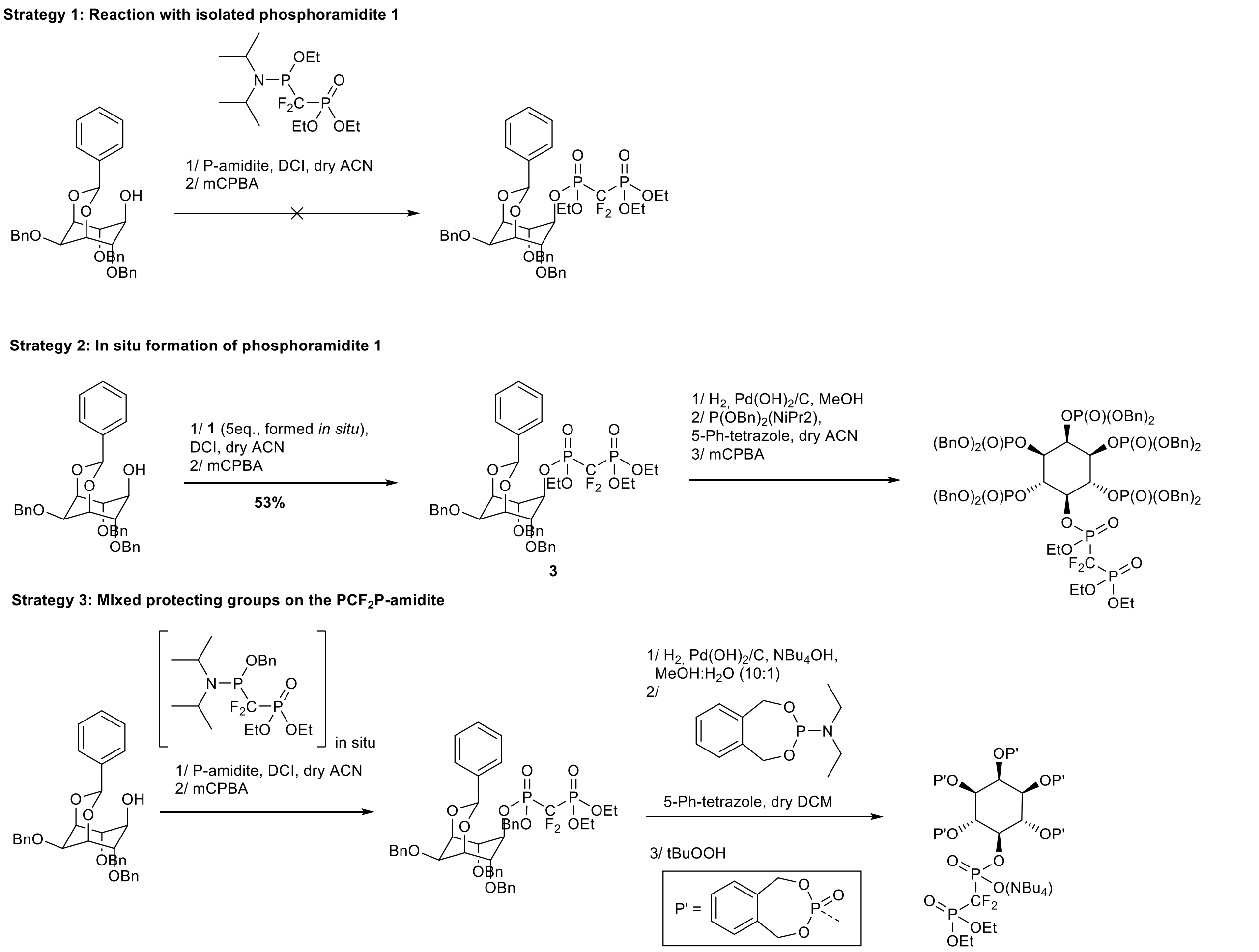

Scheme S1. Attempted synthetic routes to obtain 5PCF$_2$P-IP$_5$. We first synthesized the PCF$_2$P-amidite **2** from commercially available diethyl difluoromethyl phosphonate and N,N-diisopropylamine ethyl chlorophosphoramidite. After isolation, NMR confirmed the formation of the desired compound. However, the following reaction with inositol derivatives did not yield any product. We thus sought to form the PCF$_2$P-amidite *in situ*, and to have it react with the Ins derivative in presence of DCI, followed by oxidation of the resulting phosphate in presence of mCPBA. This strategy yielded compound **3** with moderate yields (53%). We then pursued the synthesis hydrogenation of the inositol derivative, phosphorylation of the 5 hydroxyl groups on the inositol ring and final TMS Br deprotection. However, NMR of the final compound showed unambiguously that some isomerization had occurred, and that the PCF$_2$P moiety was present not only at the 5-position but at others. We hypothesized that due to the electronegativity of the CF$_2$ moiety, the phosphorus centers of the bisphosphonate moiety were more reactive toward nucleophilic attacks by the neighboring free hydroxyl groups or 5-Ph-tetrazole. In an attempt to circumvent this, we synthesized a PCF$_2$P-amidite bearing a Bn protecting group, that would be removed during hydrogenation, leaving a deprotonated oxygen that would be less prone to reaction. Although this strategy yielded the desired compound, the final TMSBr deprotection also resulted in a mixture of compounds. Finally, we decided to append the PCF$_2$P moiety at the very end of the synthesis (see main text).

Chemical synthesis

General Information

Abbreviations

ACN	acetonitrile
DCM	dichloromethane
EA	ethyl acetate
RBF	round-bottomed flask
THF	tetrahydrofuran
TEA	triethylamine
RM	Reaction mixture

Solvents and chemicals

Commercially available chemicals were purchased from Sigma-Aldrich, Acros-Organics, Alfa-Aesar, and used as received unless otherwise stated. Dry THF and dry hexane were purchased from Acros-Organics and used as received. Dry ACN and DCM were obtained using a solvent-purification system.

Silica gel column chromatography

Automated column chromatography was carried out on normal–phase silica columns on a CombiFlash® Rf from Teledyne Isco.
For the purification of mixed P^{III}-P^{V} species, high-purity grade silica (Sigma) was used, and column chromatography was performed by hand under N_2 atmosphere using solvents containing 1% TEA.
Reactions and chromatography fractions were monitored by thin-layer chromatography using SiO_2 on aluminum plates and visualized using a 254 nm UV lamp and/or by treatment with a suitable staining solution (potassium permanganate or phosphomolybdic acid (PMA) or cerium sulfate), followed by heating.

NMR

NMR measurements of isolated compounds and quantification were performed on a Bruker AV 300, or a AV600 spectrometer.

HPLC

Preparative high-performance liquid chromatography (HPLC) was performed on a Varian system with SD-1 prep solvent delivery system, a ProStar 325 UV-Vis detector and a 440-LC fraction collector, using a Waters XBridge™ 5 µm C18 column (19 × 150 mm).

Reported compounds

Compounds **2**[1] and **4**[2] were synthesized according to published procedures, and their analytical data matched the reported values.

Compound 1

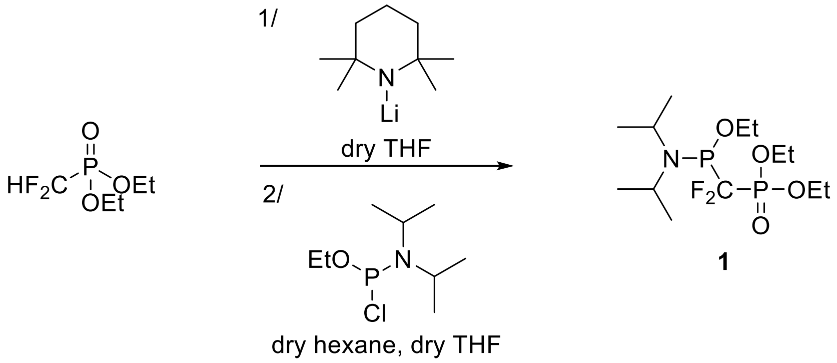

The synthesis of compound **1** was adapted from published procedures.[3–5] 2,2,6,6-tetramethylpiperidine (178 µL, 1.05 mmol) was diluted in dry THF (3mL) under a dry N_2 atmosphere. The solution was cooled to 0°C (ice bath) and a 2.5M solution of BuLi in hexanes (0.42 mL, 1.05 mmol) was added dropwise. The solution turned yellow, and was stirred for 45 min at 0°C. It was then cooled in a $CO_{2\ (s)}$-acetone bath. Diethyl (difluoromethyl) phosphonate (0.16 mL, 1.02 mmol) was added dropwise, and reaction mixture (RM) was stirred at -78°C for an hour. A solution of diethyl chlorophosphoramidite (231.6 mg, 1.09 mmol) was prepared in dry hexane (0.3 mL), filtered over a 0.45µm PTFE syringe filter and quickly added to RM at -78°C. RM was stirred for 1h30 at -78°C. Absolute EtOH (0.1 mL) was added, and RM was poured into a mixture of saturated aqueous $NaHCO_3$ (15 mL) and DCM (15 mL). After a quick extraction, the organic layer was dried over Na_2SO_4, filtered and concentrated to a yellow-orange oil. The crude material was purified by silica gel column chromatography (high-purity silica) (10% EA in hexane). A small amount of the desired product was obtained as a slightly yellow oil (11 mg, 0.030 mmol, 3 %).

^{1}H NMR (300 MHz, $CDCl_3$) δ (ppm) 4.33 – 4.21 (m, 4H, OCH_2CH_3 (P^V)), 3.89 – 3.76 (m, 2H, OCH_2CH_3 (P^{III})), 3.66 (br, 2H, $N(CH(CH_3)_2)_2$), 1.37 (t, J = 7.1 Hz, 6H, OCH_2CH_3 (P^V)), 1.29 (t, J = 7.0 Hz, 3H, OCH_2CH_3 (P^{III})), 1.18 (t, J = 7.0 Hz, 12H, 2H, $N(CH(CH_3)_2)_2$).

^{19}F NMR (282 MHz, $CDCl_3$) δ (ppm) -38.31 (ddd, $^2J_{FF}$ = 339.5 Hz, $^2J_{FP}$ = 118.7 Hz, $^2J_{FP}$ = 95.9 Hz, 1F), -48.27 (ddd, $^2J_{FF}$ = 339.5 Hz, $^2J_{FP}$ = 97.1 Hz, $^2J_{FP}$ = 54.8 Hz, 1F).

^{31}P NMR (122 MHz, $CDCl_3$) δ (ppm) 109.9 (ddd, $^2J_{PP}$ = 60.3 Hz, $^2J_{FP}$ = 118.7 Hz, $^2J_{FP}$ = 54.8 Hz, 1P, P^{III}), 8.3 (ddd, $^2J_{PP}$ = 60.3 Hz, $^2J_{FP}$ = 97.1 Hz, $^2J_{FP}$ = 95.9 Hz, 1P, P^V).

Compound 3

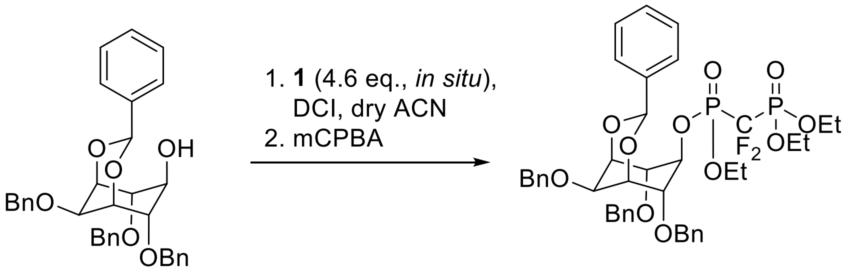

2 **3**

Anhydrous THF (4 mL) was introduced in an oven-dried 50-mL RBF under a dry N_2 atmosphere, followed by diisopropylamine (147.6 µL, 1.05 mmol). The solution was cooled in a $CO_{2(s)}$-acetone bath and degassed for 5 min under high vacuum before being restored to a dry N_2 atmosphere. A 2.5 M BuLi solution in hexanes (0.42 mL, 1.05 mmol) was added dropwise, and the reaction mixture was stirred at -78°C for 30 min. A solution of diethyl difluoromethylphosphonate (156.8 µL, 1.0 mmol) in dry THF (1 mL) was added dropwise. The resulting solution was stirred for 45-50 min at -78°C. A 0.9°M solution of diethylchlorophosphoramidite was prepared in dry hexane and filtered over a 0.45 µm PTFE syring filter, before 1.1 mL of this solution (1 mmol) were quickly added to the reaction mixture. After 2 h, the cooling bath was removed and solvents were removed by rotary evaporation. The resulting orange-brown crude was dried under high vacuum (15 min), then put under a dry N_2 atmosphere. It was redissolved in 1 mL dry ACN and cooled in an ice-brine bath. A solution of Ins derivative **2** (116 mg, 0.215 mmol) in dry acetonitrile (1.5 mL) was added at 0°C, followed by DCI (224 mg, 1.9 mmol). RM was stirred overnight, over which time it reached RT. A solution of mCPBA (340 mg, 1.52 mmol) in dry ACN (1 mL) was added dropwise at -20°C, and RM was stirred for 1h30 min at -20°C then 30 min at RT. RM was diluted with sat. aq. $Na_2S_2O_3$ (30 mL) and extracted with EA (30 mL). the organic layer was then washed with sat. aq. $NaHCO_3$ (30 mL) and brine (30 mL). The organic layer was dried over Na_2SO_4, filtered and evaporated. It was purified by silica gel flash column chromatography (25 to 50% EA in hexane), to yield the desired compound as a white solid (98.6 mg, 0.121mmol, 56%).

^{1}H NMR (300 MHz, $CDCl_3$) δ (ppm) 7.64 – 7.55 (m, 2H), 7.48 – 7.27 (m, 18H), 5.87 (s, 1H), 4.83 – 4.64 (m, 7H), 4.45 (d, J = 2.3 Hz, 2H), 4.32 (ddt, J = 15.8, 12.9, 5.2 Hz, 7H), 4.12 (dt, J = 10.1, 6.9 Hz, 1H), 3.65 (t, J = 2.3 Hz, 1H), 1.38 (td, J = 7.1, 4.0 Hz, 6H), 1.17 (td, J = 7.1, 0.7 Hz, 3H).

^{13}C NMR (300 MHz, $CDCl_3$) 137.87, 137.71, 137.26, 137.20, 129.52, 128.55, 128.46, 128.45, 128.34, 127.93, 127.91, 127.85, 127.75, 126.60, 93.14, 80.96, 80.93, 80.65, 80.58, 80.54, 73.35, 73.14, 71.96, 71.89, 70.93, 67.69, 66.08 (d, J = 6.0 Hz), 65.50 (d, J = 6.2 Hz), 65.39 (d, J = 6.2 Hz), 16.45 (d, J = 5.2 Hz), 15.93 (d, J = 6.0 Hz). The CF_2 signal was not detected.

^{19}F NMR (282 MHz, $CDCl_3$) δ (ppm) -43.92, -43.50 (AB_q, 2F, $^2J_{PF}$ = 86.3 Hz).

^{31}P NMR (122 MHz, $CDCl_3$) δ (ppm) 2.66, 3.62 (AB_q, 2P, $^2J_{PF}$ = 86.3 Hz).

HRMS (ESI): calculated for $C_{41}H_{48}F_2O_{11}P_2Na^+$: 840.2566 $[M+Na]^+$, found 840.2621.

Compound 5

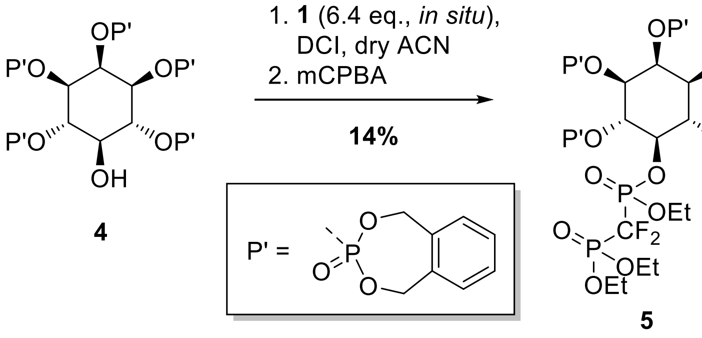

Diisopropylamine (147 µL, 1.05 mmol) was dissolved in 4 mL dry THF under a dry N_2 atmosphere. The solution was cooled in a $CO_{2(s)}$-acetone bath, and degassed under high vacuum, before being restored to a dry N_2 atmosphere. A 2.5 M BuLi solution in hexanes (0.42 mL, 1.05 mmol) was added dropwise, and the reaction mixture was stirred at -78°C for 30 min. A solution of diethyl difluoromethylphosphonate (156.8 µL, 1.0 mmol) in dry THF (1 mL) was added dropwise. The resulting pale yellow solution was stirred for 40-50 min at -78°C. A 1°M solution of diethylchlorophosphoramidite was prepared in dry hexane and filtered, before 1 mL of this solution (1 mmol) added quickly to reaction mixture. After 2 h, the cooling bath was removed and solvents were removed under reduced pressure. The resulting orange-brown crude was dried under high vacuum (15 min), then put under a dry N_2 atmosphere. A solution of Ins derivative (170 mg, 0.156 mmol) in dry acetonitrile (3 mL) was added at 0°C, followed by DCI (336 mg, 2.8 mmol). RM was stirred overnight at 0°C to RT. mCPBA (334 mg, 1.5 mmol) was added portionwise at 0°C, and RM was stirred for 20-30 min at 0°C then 15-20 min at RT. RM was diluted with EA (25 mL) and washed with sat. aq. $Na_2S_2O_3$ (20 mL), sat. aq. $NaHCO_3$ (20 mL) and brine (20 mL). The organic layer was dried over Na_2SO_4, filtered and evaporated. It was purified by silica gel flash column chromatography (0 to 10% MeOH in DCM), then by preparative HPLC to yield the desired compound as a white solid (28 mg, 0.021mmol, 14%).

Preparative HPLC conditions: XBridge. A = water, no TFA. B = ACN, no TFA. 30 mL/min. 2 min 50%B, then 50 to 70%B in 4 min, then 70%B for 2 min. detection 214 nm. Product @ 4.4 min, starting material @ 3.6 min).

^{1}H NMR (600 MHz, $CDCl_3$) δ (ppm) 7.34 – 7.23 (m, 20H), 5.73 (m, 2H), 5.67 (m, 2H), 5.56 (m, 1H), 5.53 – 5.43 (m, 6H), 5.24 – 5.04 (m, 12H), 4.98 (m, 2H), 4.88 (m, 1H), 4.44 (m, 2H), 4.34 (m, 4H), 1.36 (m, 9H).

^{13}C NMR (151 MHz, $CDCl_3$) δ (ppm) 135.7, 135.6, 135.5, 135.5, 135.4, 135.0, 134.9, 129.6, 129.5, 129.5, 129.3, 129.3, 129.2, 129.1, 129.1, 129.0, 128.8, 128.7, 76.2, 75.6, 73.9, 73.8, 69.56 (m), 69.25 (m), 67.3, 65.75 (d, J = 2.9 Hz), 65.58 (d, J = 3.2 Hz), 16.5 (br), 16.16 (d, J = 3.2 Hz). The CF_2 was not detected.

^{19}F NMR (565 MHz, $CDCl_3$) δ (ppm) 1.57 (m).

^{31}P NMR (243 MHz, $CDCl_3$) δ (ppm) 0.4 (td, J_{PF} = 86.9 Hz, J_{PP} = 9.1 Hz, 2P), - 5.0 (d, J = 18 Hz, 2P), - 6.42 (s, 1P), - 6.52(s, 1P), - 6.69 (s, 1P).

Compound 5PCF$_2$P-IP$_5$

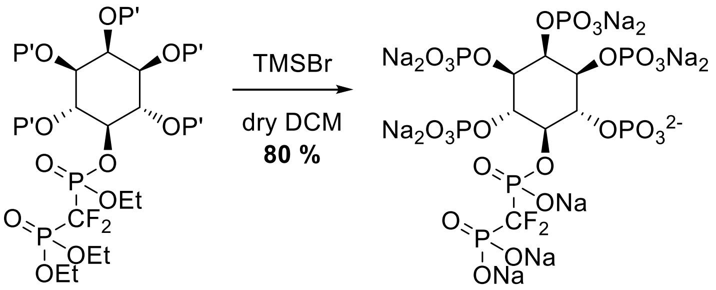

5 **5PCF$_2$P-IP$_5$**

Compound **5** (19 mg, 14 µmol) was dissolved in dry DCM (750 µL) under a N_2 atmosphere. TMSBr (250 µL) was added at 0°C, and the reaction mixture was left to reach RT and to stir for 6 h. Solvents were removed by rotary evaporation, and 3 mL MeOH were added to the resulting residue. Solvent was again removed by rotary evaporation. The crude residue was taken up in Et_2O (3 mL) and 1M TEAB (3 mL). The aqueous layer was washed once more with Et_2O (3 mL), then coevaporated several times with MeOH, and lyophilized The resulting crude was purified by anion exchange chromatography on a 20 mL Q H column (A: water, B: 1M NH_4HCO_3 pH 7.6). Fractions containing the desired compound were lyophilized and pooled. The resulting white solid was finally redissolved in water and stirred overnight with a Chelex resin. The flowthrough was lyophilized, yielding 11 mg of the desired compound as a white solid.

^{1}H NMR (600 MHz, D_2O, pH^{app} = 6) δ (ppm) 4.63 (d, J = 9.5 Hz, 1H), 4.33 (q, J = 9.5 Hz, 2H), 4.15 (q, J = 9.1 Hz, 1H), 3.99 (t, J = 9.0 Hz, 2H).

^{19}F NMR (564 MHz, D_2O, pH^{app} = 6) δ (ppm) – 42.36 (br).

^{31}P NMR (243 MHz, D_2O, pH^{app} = 6) δ (ppm) 4.93 (m, 1P), 0.21 (m, 1P), 1.98 (s, 2P), -2.46 (s, 2P), -2.81 (s, 1P).

Biochemical assays

Protein expression and purification.

Throughout the main text, PPIP5K2^{KD} is deployed as an abbreviation for the human PPIP5K2 kinase domain. However, two different constructs were utilized, as described in the figure legends and within the Methods section below: one comprised residues 1–366 (PPIP5K2$^{1\text{-}366}$) and the other comprised residues 41–366 (PPIP5K2$^{41\text{-}366}$). Both constructs were expressed and purified as described previously.[6] The human DIPP1 construct (residues 1-148) was expressed and purified as described in [7].

Crystallization and structure determination

PPIP5K2$^{41\text{-}366}$ was crystallized as previously described.[6] The crystals were incubated for three days in a soaking buffer containing 2 mM 5-PCF$_2$P-IP$_5$, 22% (w/v) PEG 3350, 10 mM $MgCl_2$, 0.1 M sodium acetate, pH 5.2 at 4 °C and the crystals were then soaked under the above stabilizing buffer for three days with. Prior to freezing, 33% ethylene glycol was added to the soaking buffer.
The catalytic domain of human DIPP1 (residues 1-148) was crystallized by incubating 11.2 mg/mL of DIPP1 protein plus 2 mM ligand on ice for 30 minutes. Crystals were formed by the hanging drop vapor diffusion method; hanging drops comprised 1.5 µL of protein/ligand solution plus 1.5 µL of the “co-crystallization medium” that was present in the reservoir, which comprised 10% (w/v) PEG 8000, 10% (v/v) isopropanol, 200 mM Li_2SO_4, 75 mM Na acetate and 25 mM HEPES, final pH 5.9, plus 2 mM ligand. Prior to freezing, the isopropanol concentration was increased to 20%.
Diffraction data were collected using APS beamlines 22-ID. All data were processed with the program HKL2000. The structure was determined using rigid body and direct Fourier synthesis, and refined with the equivalent and expanded test sets. The structure was further manually rebuilt with COOT and refined with REFMAC from the CCP4 package. The molecular graphics representations were prepared with the program PyMol (Schrödinger, LLC). Atomic coordinates and structure factors have been deposited with the Protein Data Bank with accession codes 8G9C, 8G9D, 8G9E.

PPIP5K2^{KD} reverse kinase assay

For the reverse kinase assay, PPIP5K2$^{1\text{-}366}$ (2.5 µg mL^{-1}) was incubated at 25 °C for 30 min with 20 µL buffer containing 20 mM Tris–HCl, pH 7.5, 10 mM $MgCl_2$, 0.1 mM ADP, 100 nM [1,5]PP-IP$_4$ and various concentrations of either 5PCP-IP$_5$ or 5PCF$_2$P-IP$_5$. The generated ATP was measured using a Molecular Probes ATP Determination kit (Thermo Fisher Scientific catalog number A22066). The IC_{50} value was calculated using GraphPad Prism.

Isothermal titration calorimetry

Calorimetry experiments were performed using a MicroCal PEAQ-ITC (Malvern Panalytical) with 7.5 µM recombinant PPIP5K2$^{1\text{–}366}$ in the sample cell and 75 µM of ligand in the syringe, each of which was maintained at 25°C in buffer containing 20 mM HEPES, pH 7.2, 150 mM KCl, 0.05 mM EDTA, 1mM AMP-PNP, 1mM $MgCl_2$ as indicated. The sample cell (volume = 204 µL) and the syringe were cleaned before each run. Thermograms were constructed from 13 injections, each of which involved 3 µL of ligand delivered for 6 s, with an equilibration time of 150-300 s between each injection. The stirring speed was set to 750 rpm. Data were fitted to a single binding site model using the analysis software provided by the manufacturer. At least three runs were performed for each condition.

HPLC Analysis

Kinase activity was studied at 37°C by incubating 700 ng PPIP5K2$^{1\text{–}366}$ separately with each individual 10 µM test compound and 50 µM ATP plus 60,000 CPM of [^{33}P-γ]ATP for 30 mins. Assays were quenched with 0.2 volumes of 2 M perchloric acid, neutralized, and [^{33}P]-labeled products were analyzed by ion-exchange HPLC, using a 4.6 × 125 mm, 5 µm PartiSphere SAX column. The elution gradient (1 mL/min) was generated by mixing Buffer A (1 mM Na_2EDTA) with Buffer B (Buffer A plus 2.5 M $NH_4H_2PO_4$, pH 4.0); the elute was mixed with 2.5 ml/min Monoflow4 scintillation liquid (National Diagnostics) and radioactivity was monitored with an in-line counter.

Author Contributions

SH: Conceptualization, Investigation, Methodology, Validation, Visualization, Writing – original draft, Writing – review & editing
HW: Conceptualization, Investigation, Methodology, Validation, Visualization, Writing – original draft, Writing – review & editing.
GZ: Investigation.
KF: Resources
AMR: Resources, Writing – review & editing
BVLP: Resources, Funding acquisition, Writing – review & editing
SBS: Conceptualization, Funding acquisition, Project administration, Writing – original draft, Writing – review & editing
DF: Conceptualization, Funding acquisition, Project administration, Writing – original draft, Writing – review & editing

References

[1] C. Murali, M. S. Shashidhar, C. S. Gopinath, *Tetrahedron* **2007**, *63*, 4149–4155.
[2] H. Zhang, J. Thompson, G. D. Prestwich, *Org. Lett.* **2009**, *11*, 1551–1554.
[3] G. K. S. Prakash, M. Zibinsky, T. G. Upton, B. A. Kashemirov, C. E. McKenna, K. Oertell, M. F. Goodman, V. K. Batra, L. C. Pedersen, W. A. Beard, D. D. Shock, S. H. Wilson, G. A. Olah, *Proc. Natl. Acad. Sci.* **2010**, *107*, 15693–15698.
[4] S. B. Engelsma, N. J. Meeuwenoord, H. S. Overkleeft, G. A. van der Marel, D. V. Filippov, *Angew. Chem. Int. Ed.* **2017**, *56*, 2955–2959.
[5] S. Hostachy, T. Utesch, K. Franke, G. L. Dornan, D. Furkert, B. Türkaydin, V. Haucke, H. Sun, D. Fiedler, *Chem. Sci.* **2021**, *12*, 10696–10702.
[6] H. Wang, J. R. Falck, T. M. T. Hall, S. B. Shears, *Nat. Chem. Biol.* **2012**, *8*, 111–116.
[7] G. Zong, N. Jork, S. Hostachy, D. Fiedler, H. J. Jessen, S. B. Shears, H. Wang, *FASEB J.* **2021**, *35*, e21275.

Appendices: NMR spectra

^{1}H NMR spectrum of compound 1

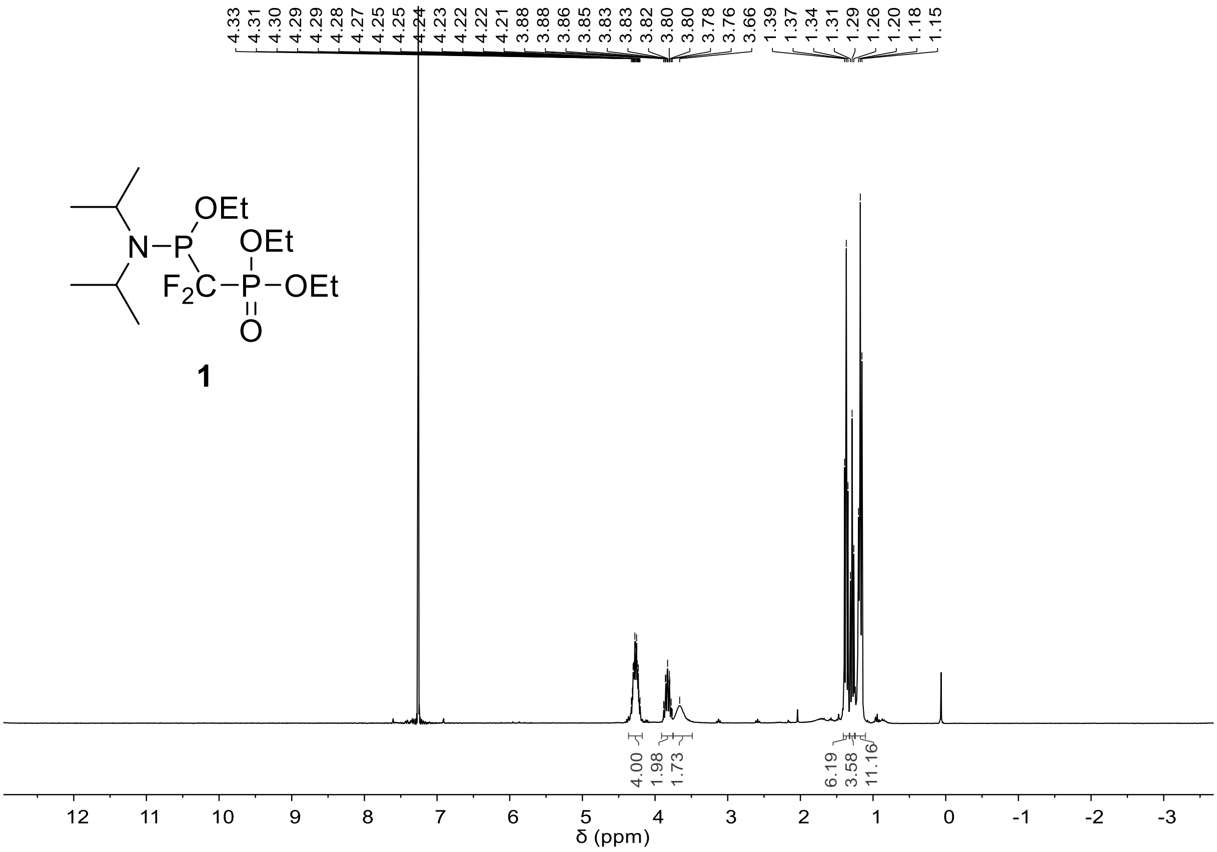

^{19}F NMR spectrum of compound 1

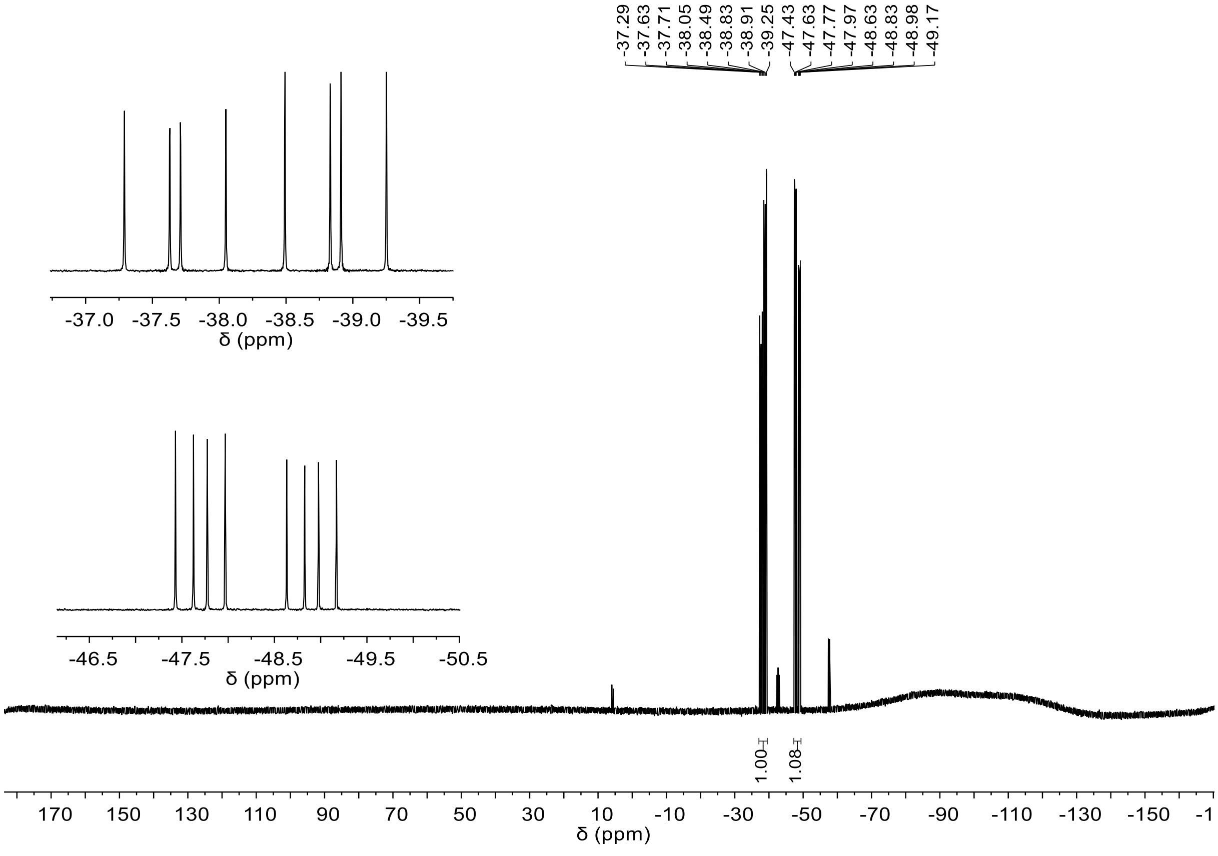

^{31}P NMR spectrum of compound 1

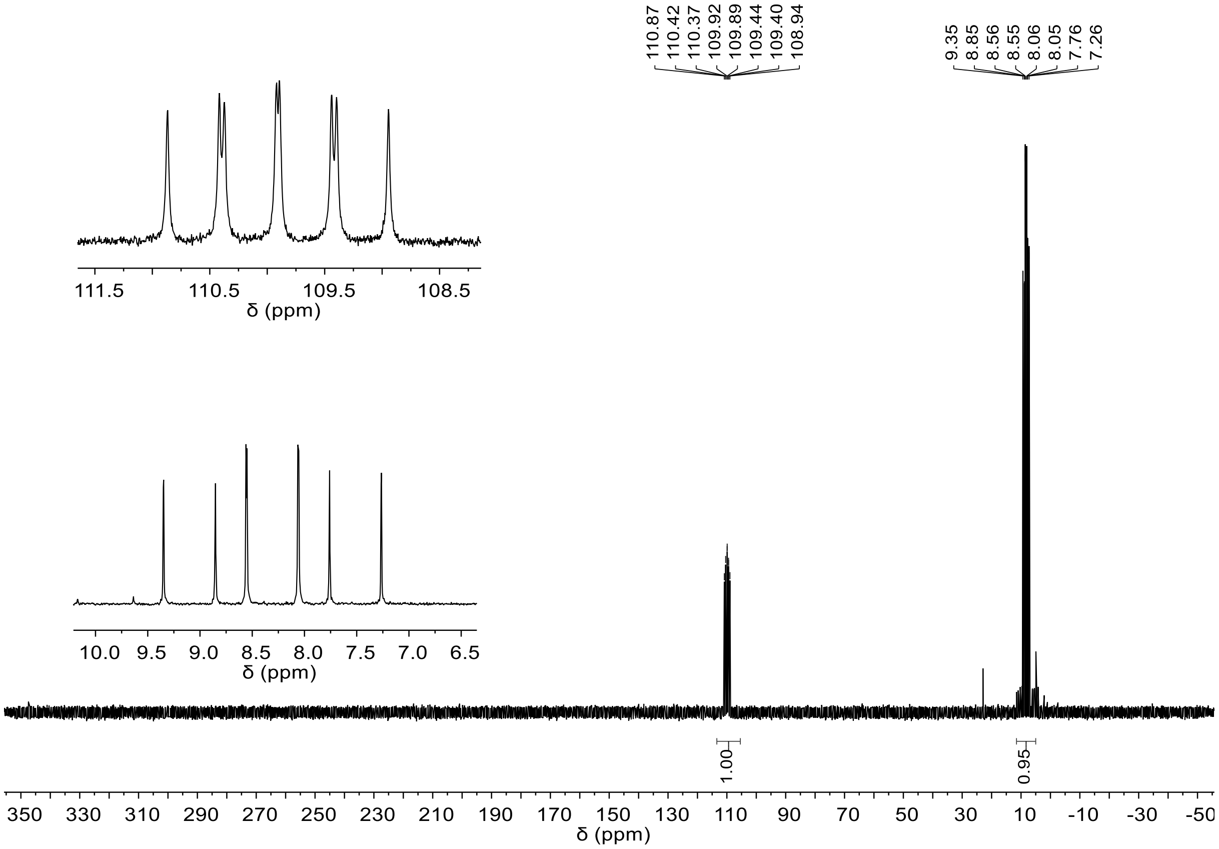

^{1}H NMR spectrum of compound 3

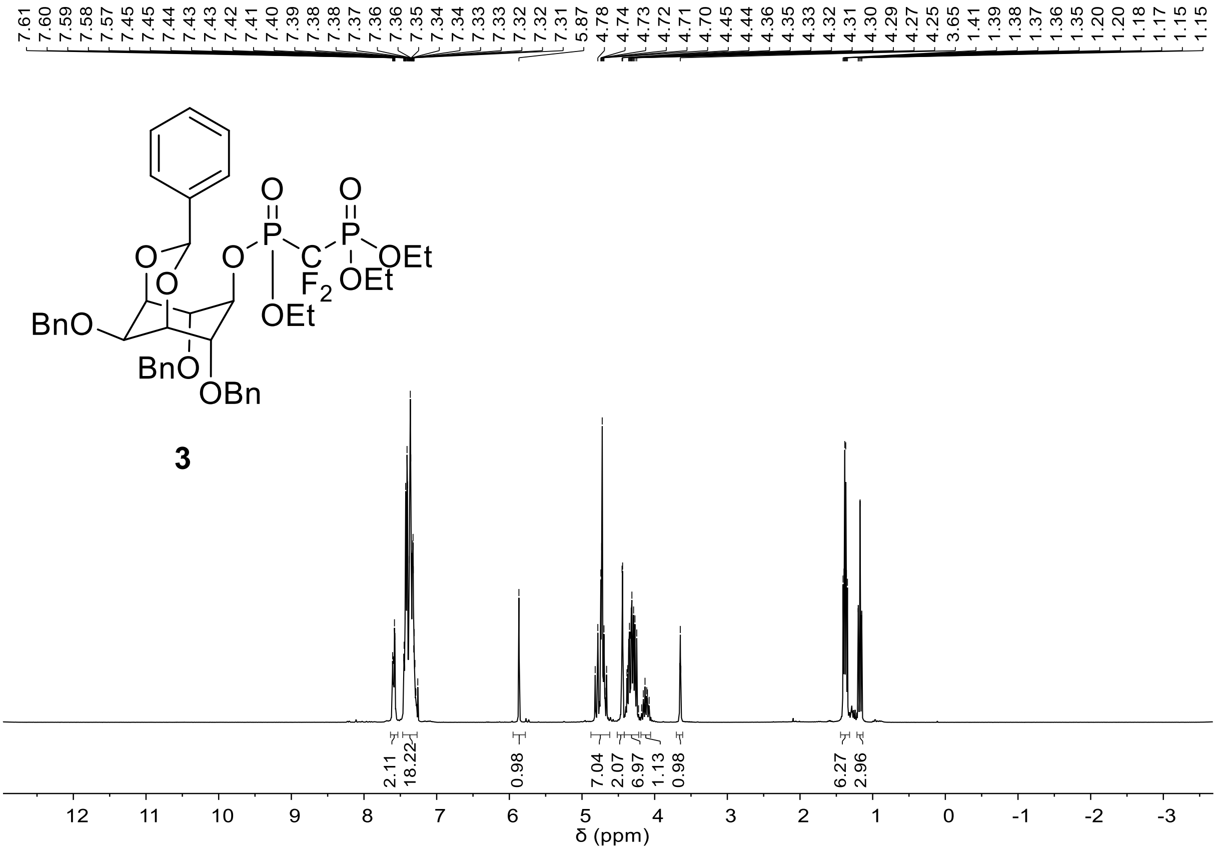

^{13}C NMR spectrum of compound 3

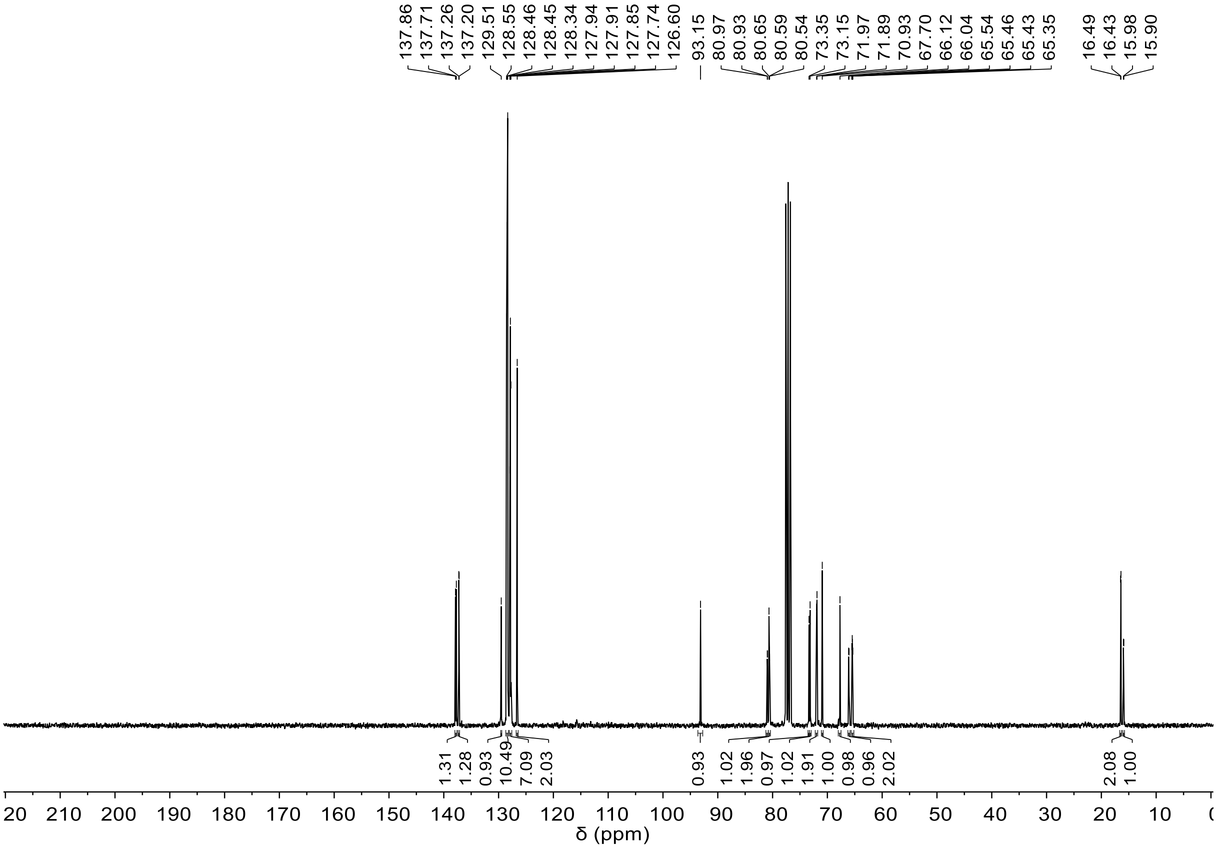

^{19}F NMR spectrum of compound 3

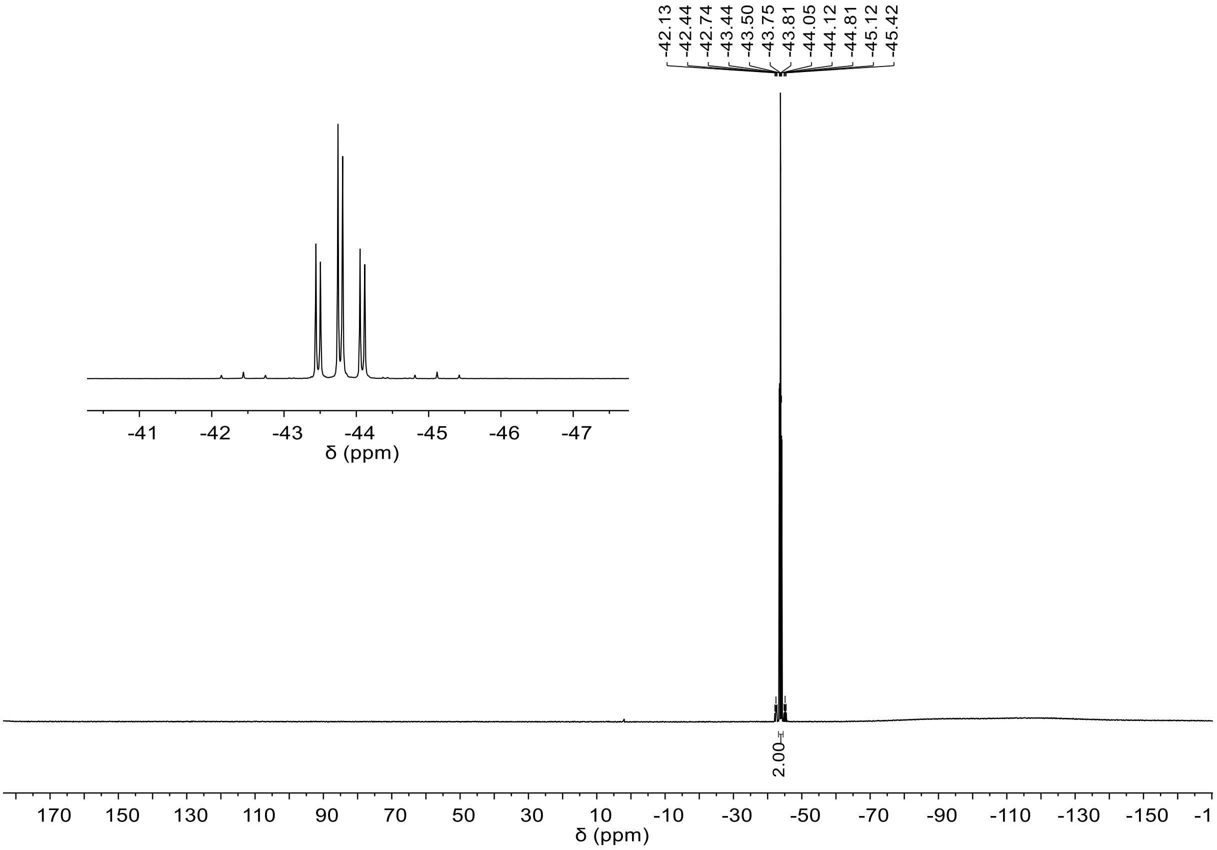

^{31}P NMR spectrum of compound 3

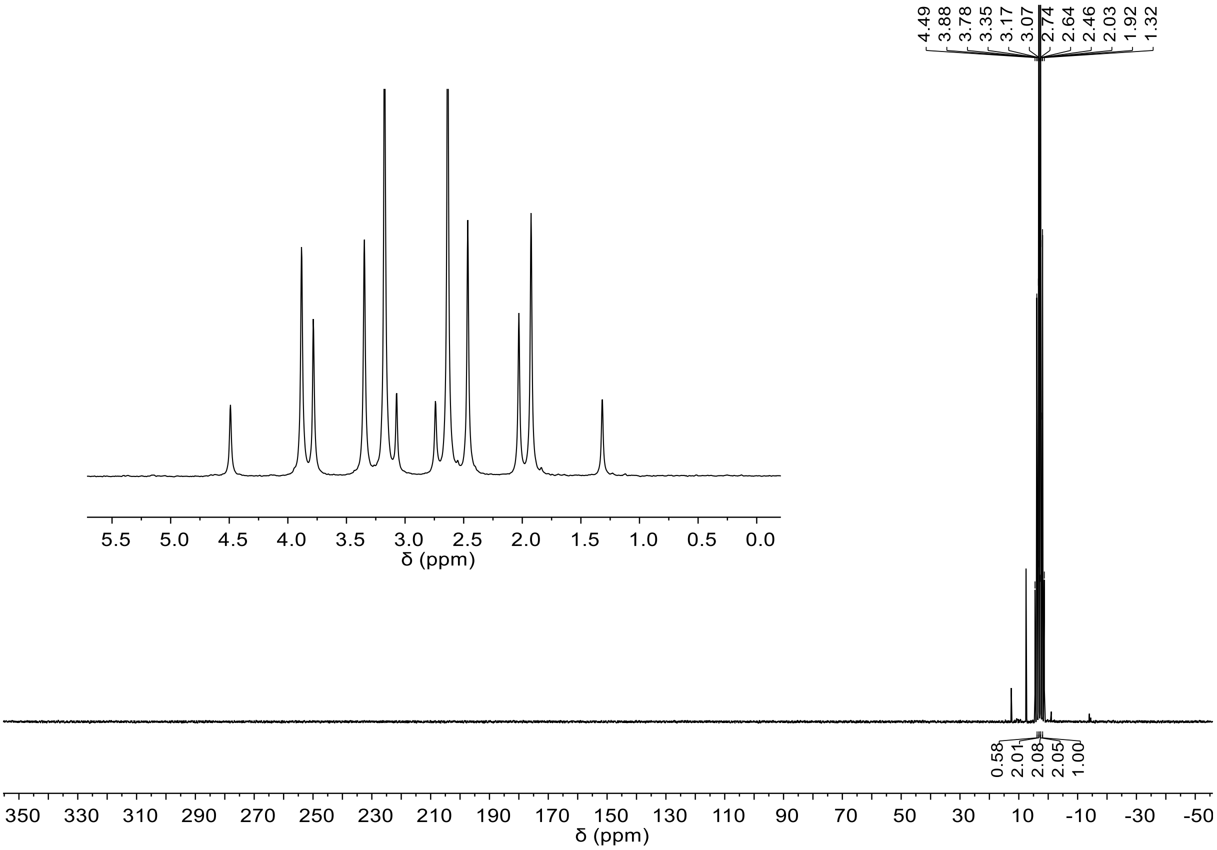

^{1}H NMR spectrum of compound 5

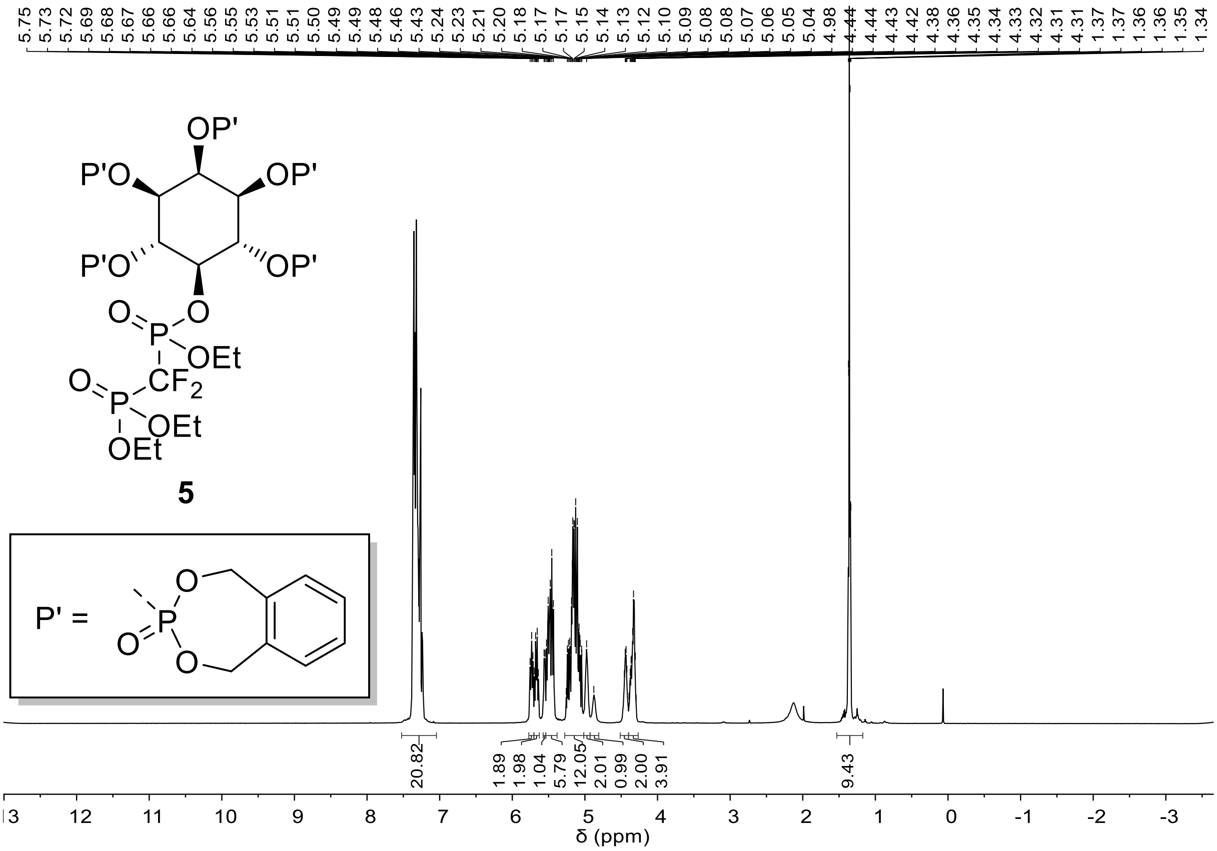

^{13}C NMR of compound 5

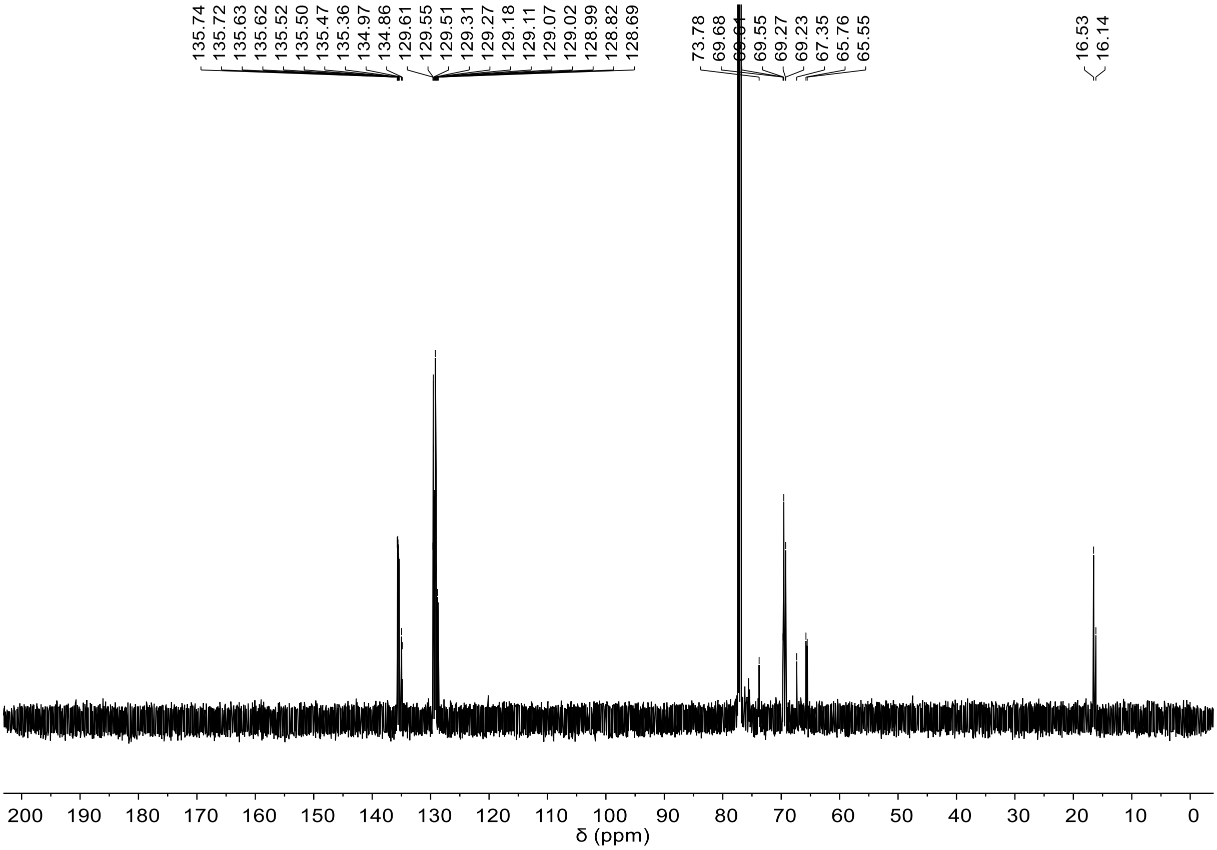

^{19}F NMR spectrum of compound 5

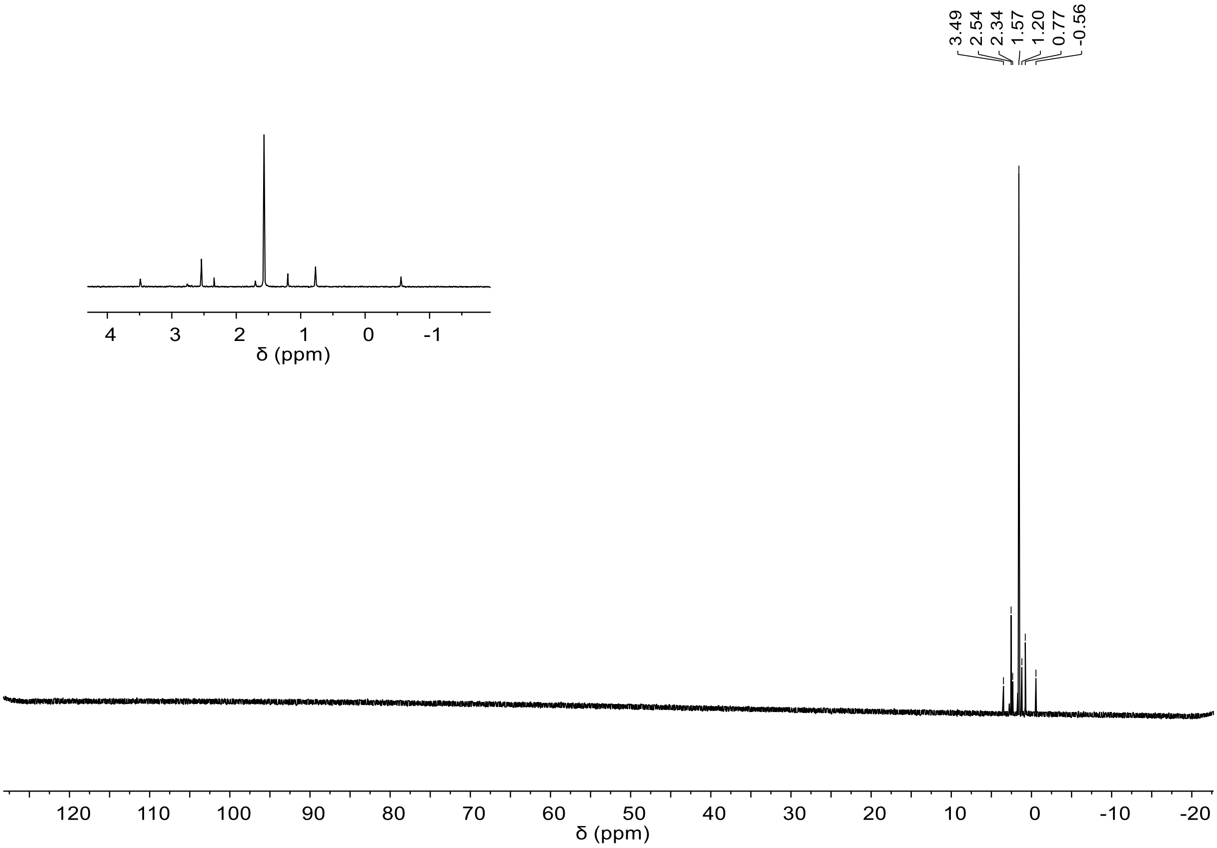

^{31}P NMR spectrum of compound 5

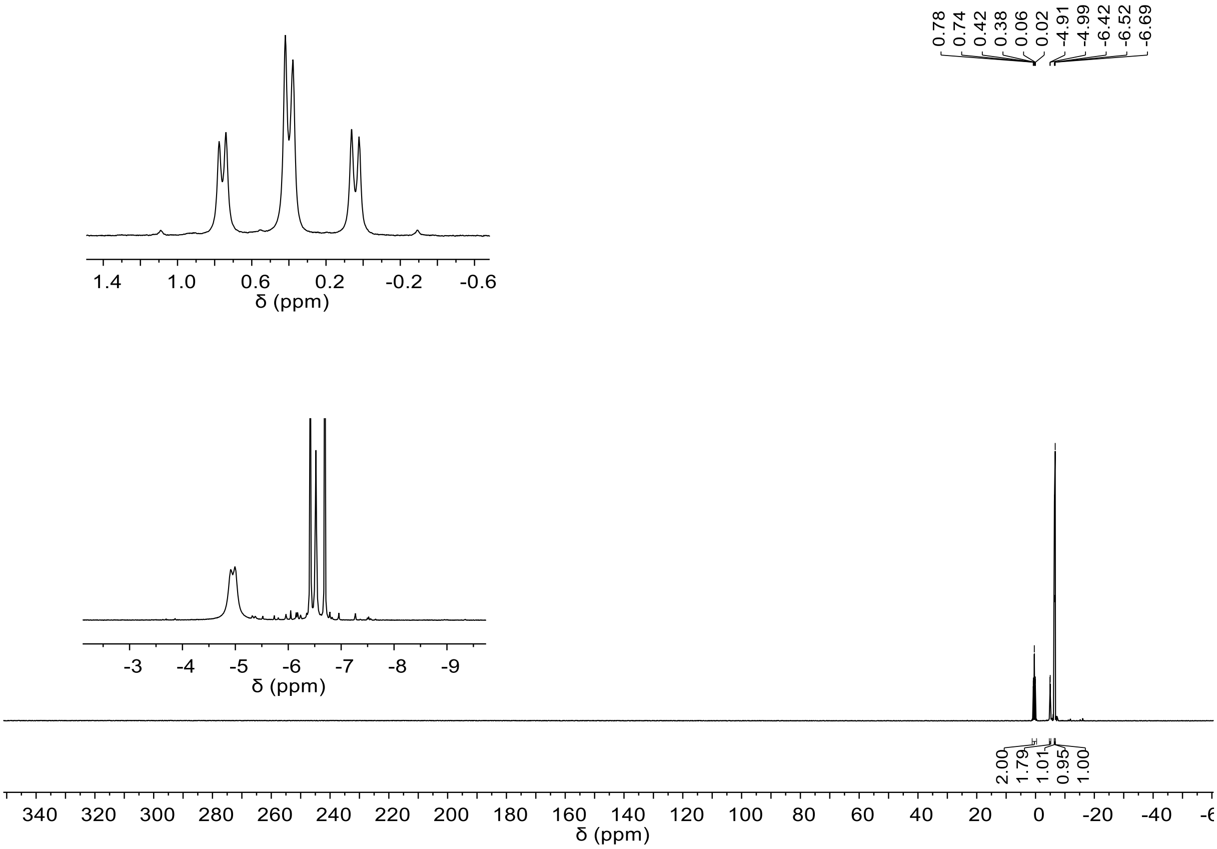

^{1}H NMR spectrum of 5PCF$_2$P-IP$_5$

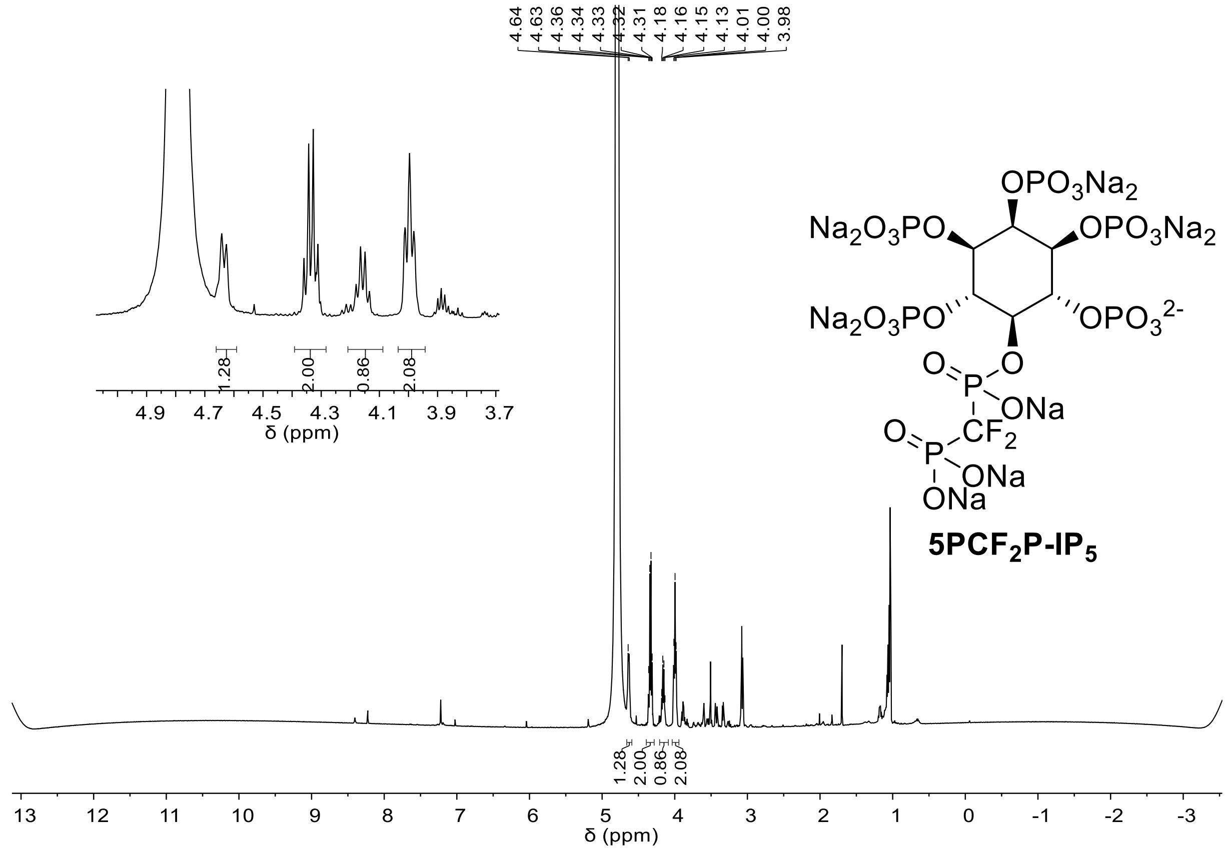

^{19}F NMR spectrum of 5PCF$_2$P-IP$_5$

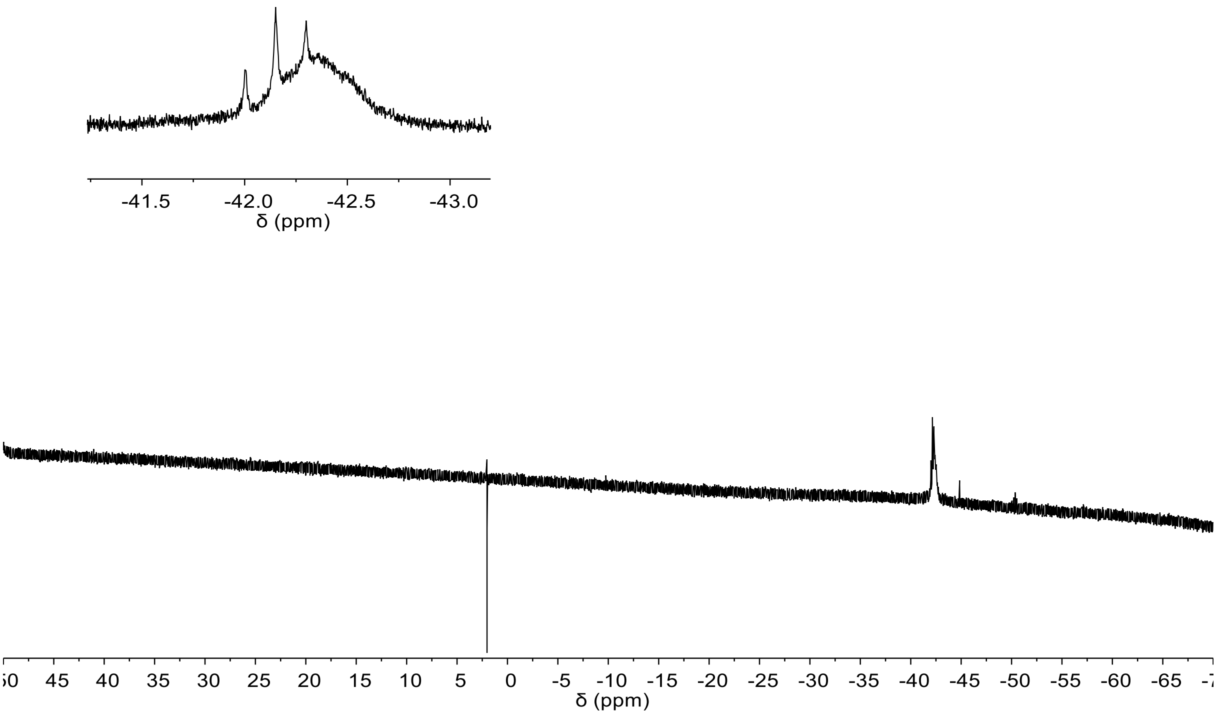

^{31}P NMR spectrum of 5PCF$_2$P-IP$_5$

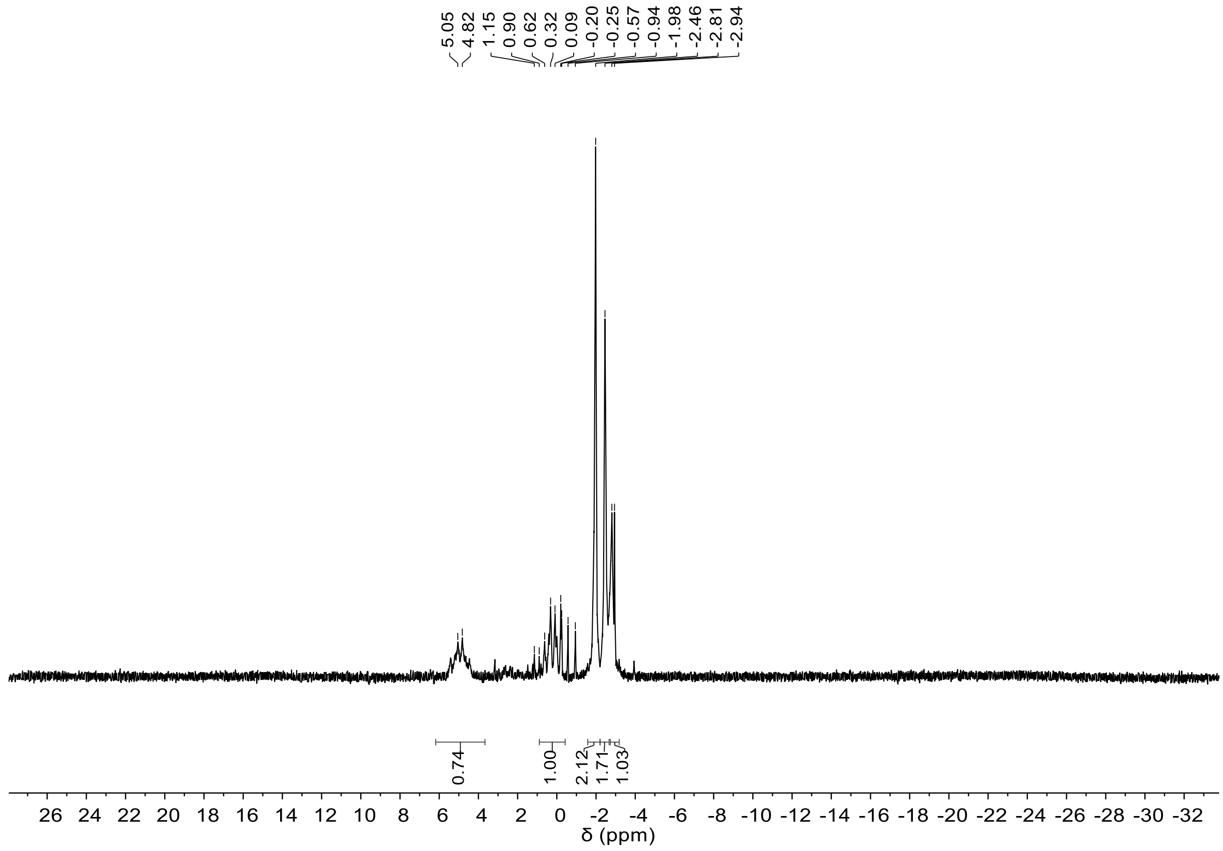

Supplement: Supplementary file 1 [file CHEM-29-0-s001.pdf]
